# Enhancing Human Health Through Nutrient and Bioactive Compound Recovery from Agri-Food By-Products: A Decade of Progress

**DOI:** 10.3390/nu17152528

**Published:** 2025-07-31

**Authors:** Cinzia Ingallina, Mattia Spano, Sabrina Antonia Prencipe, Giuliana Vinci, Antonella Di Sotto, Donatella Ambroselli, Valeria Vergine, Maria Elisa Crestoni, Chiara Di Meo, Nicole Zoratto, Luana Izzo, Abel Navarré, Giuseppina Adiletta, Paola Russo, Giacomo Di Matteo, Luisa Mannina, Anna Maria Giusti

**Affiliations:** 1 Department of Chemistry and Technology of Drugs, Sapienza University of Rome, 00185 Rome, Italy; cinzia.ingallina@uniroma1.it (C.I.); donatella.ambroselli@uniroma1.it (D.A.); valeria.vergine@uniroma1.it (V.V.); mariaelisa.crestoni@uniroma1.it (M.E.C.); chiara.dimeo@uniroma1.it (C.D.M.); giacomo.dimatteo@uniroma1.it (G.D.M.); luisa.mannina@uniroma1.it (L.M.); 2 Department of Management, Sapienza University of Rome, Via del Castro Laurenziano 9, 00161 Rome, Italy; sabrinaantonia.prencipe@uniroma1.it (S.A.P.); giuliana.vinci@uniroma1.it (G.V.); 3 Department of Physiology and Pharmacology ‘V. Erspamer’, Sapienza University of Rome, 00185 Rome, Italy; antonella.disotto@uniroma1.it; 4 School of Pharmacy, University of Rome “Tor Vergata”, 00133 Rome, Italy; nicole.zoratto@uniroma2.it; 5 Department of Pharmacy, Faculty of Pharmacy, University of Naples “Federico II”, Via Domenico Montesano 49, 80131 Naples, Italy; luana.izzo@unina.it (L.I.);; 6 Department of Chemical Engineering Materials Environment, Sapienza University of Rome, 00184 Rome, Italy; giuseppina.adiletta@uniroma1.it (G.A.); paola.russo@uniroma1.it (P.R.); 7 Department of Experimental Medicine, Sapienza University of Rome, 00161 Rome, Italy

**Keywords:** agri-food waste, by-products, valorization, bioactive compounds, antioxidant/biological activity, functional foods, nutraceuticals, human health, innovative extraction techniques, sustainability

## Abstract

In light of pressing global nutritional needs, the valorization of agri-food waste constitutes a vital strategy for enhancing human health and nutrition, while simultaneously supporting planetary health. This integrated approach is increasingly indispensable within sustainable and equitable food systems. Recently, a sustainability-driven focus has shifted attention toward the valorization of the agri-food by-products as rich sources of bioactive compounds useful in preventing or treating chronic diseases. Agri-food by-products, often regarded as waste, actually hold great potential as they are rich in bioactive components, dietary fiber, and other beneficial nutrients from which innovative food ingredients, functional foods, and even therapeutic products are developed. This review aims to provide a comprehensive analysis of the current advances in recovering and applying such compounds from agri-food waste, with a particular focus on their roles in human health, sustainable packaging, and circular economy strategies. **Methods**: This review critically synthesizes recent scientific literature on the extraction, characterization, and utilization of bioactive molecules from agri-food by-products. After careful analysis of the PubMed and Scopus databases, only English-language articles from the last 10 years were included in the final narrative review. The analysis also encompasses applications in the nutraceutical, pharmaceutical, and food packaging sectors. **Results**: Emerging technologies have enabled the efficient and eco-friendly recovery of compounds such as polyphenols, carotenoids, and dietary fibers that demonstrate antioxidant, antimicrobial, and anti-inflammatory properties. These bioactive compounds support the development of functional foods and biodegradable packaging materials. Furthermore, these valorization strategies align with global health trends by promoting dietary supplements that counteract the effects of the Western diet and chronic diseases. **Conclusions**: Valorization of agri-food by-products offers a promising path toward sustainable development by reducing waste, enhancing public health, and driving innovation. This strategy not only minimizes waste and supports sustainability, but also promotes a more nutritious and resilient food system.

## 1. Introduction

Given current global nutritional demands, the valorization of agri-food waste emerges not only as a beneficial strategy for enhancing human nutrition and health but also for ensuring sustainability, resource efficiency, and equitable access to nutrients in a rapidly changing food system.

In recent years, the global agri-food industry has faced growing concerns related to sustainability and environmental protection due to the massive generation of by-products and waste during food processing; an amount of about 59 million tons is produced each year in Europe alone [1]. These agri-food by-products and waste represent a significant environmental burden, contributing to greenhouse gas emissions, soil and water pollution, and resource depletion [2,3].

Nowadays, the growing focus on sustainability and the circular economy has highlighted the strategic potential of food by-products. Their valorization has emerged as a promising strategy aiming to minimize waste generation, recover valuable compounds, and mitigate the environmental impact of food processing activities [4].

Given the significant quantities of by-products from the agri-food industry, traditionally considered waste, there is growing scientific interest in developing new advanced approaches for their effective valorization that involve the transformation of waste into high-value-added raw materials by recognizing them as valuable sources of bioactive compounds characterized by several potential activities. In particular, polyphenols, carotenoids, dietary fibers, essential oils, organic acids, and bioactive peptides have been found in agri-food by-products, underlining their potential to be used for several applications, namely functional foods, nutraceuticals, cosmetics, pharmaceutical applications, and biodegradable packaging [2,5,6,7,8]. This strategy not only reduces environmental impact but also fosters the creation of new economic opportunities and drives technological innovation, promoting also a more nutritious and resilient food system.

Innovative applications include their use as ingredients in functional foods and dietary supplements capable of supporting wellness and preventing chronic diseases [9,10]. Advanced biotransformation technologies further enhance the bioaccessibility of such compounds, allowing for their integration into diets and personalized nutrition strategies aimed at improving public health [11]. As part of an integrated food system approach, the exploitation of these by-products is in line with the objectives of sustainable nutrition and could contribute to addressing diet-related non-communicable diseases by promoting high-quality food solutions with particular emphasis on the qualitative properties of nutrients and the profile of bioactive compounds [12], ultimately offering enhanced benefits for human health.

The rapid social, economic, and cultural changes that have affected the history of industrialized countries over the past 60 years have led to a profound shift in lifestyles, dietary habits, and nutrition, with the consequent prevalence of a dietary pattern known as the “Western diet”, characterized by energy-dense foods rich in saturated fats and simple sugars. Combined with a more sedentary lifestyle, this dietary pattern predisposes individuals to an increased risk of chronic degenerative diseases [13]. Therefore, the isolation of functional molecules derived from food industry waste and by-products could serve as a valuable source of bioactive compounds for the development of dietary supplements [14]. When appropriately integrated into a balanced diet, they can contribute to the maintenance of an optimal health status. In light of the growing consumer demand for healthy foods, both the industry and the scientific community have initiated the development of new ingredients for food and beverages. In this context, the integration of bioactive components—such as polyphenols, vitamins, peptides, and lipids—into diets could play a decisive role in preventing chronic degenerative diseases [15].

In the formulation of nutraceuticals, functional foods, and other value-added products derived from by-products/waste, extraction techniques used to recover bioactive compounds from agri-food processing waste play a crucial role. In recent years, a range of advanced and sustainable extraction technologies have been introduced, including ultrasound-assisted extraction, supercritical fluid extraction, enzyme-assisted processes, and fermentation-based methods. These approaches offer greater efficiency and environmental compatibility, as they minimize the need for harmful solvents and reduce energy consumption [16].

Moreover, the application of these bioactive compounds in the development of bio-based materials for sustainable packaging has gained significant attention for eco-friendly alternatives to petroleum-based plastics, thus supporting a reduction in plastic waste and enhancing resource efficiency [17,18].

Finally, the impact of agri-food waste valorization on sustainable development goals is evident across multiple dimensions. It enhances waste management and minimizes environmental impact, supporting public health, stimulating innovation, and encouraging inclusive economic growth. Integrating these strategies into industrial and environmental policies is essential for aligning with sustainable development goals, paving the way for a future where the valorization of food by-products becomes a cornerstone of a new green economy [3].

This review aims to provide a comprehensive analysis of the latest advances in the valorization of agri-food by-products, focusing on the extraction and application of bioactive compounds for the formulation of nutraceuticals/supplements and functional foods with the aim of improving health and preventing chronic degenerative diseases. Furthermore, the use of by-products for sustainable bio-based packaging development was explored. By highlighting the most recent scientific achievements and identifying current challenges, this work seeks to take into consideration future research and industrial strategies aimed at enhancing human and environmental well-being through the sustainable management of agri-food waste.

## 2. Valorization of Agri-Food Waste and By-Products in the Context of Sustainable Development Goals (SDGs)

To conduct the literature review about agri-food waste valorization in the context of SDGs, the following keywords were used: “food waste”, valorization strategy”, “SDGs”, “sustainable nutrition”, “circular economy”, “food market”, “food waste recovery”, “food category”, “essential biomolecules”, and “bioactive compounds”. This research focused on original articles—excluding reviews—published in the last ten years, using bibliographic databases such as Scopus, Web of Science, Google Scholar, and PubMed. Only peer-reviewed articles in English were used.

**Figure 1:**
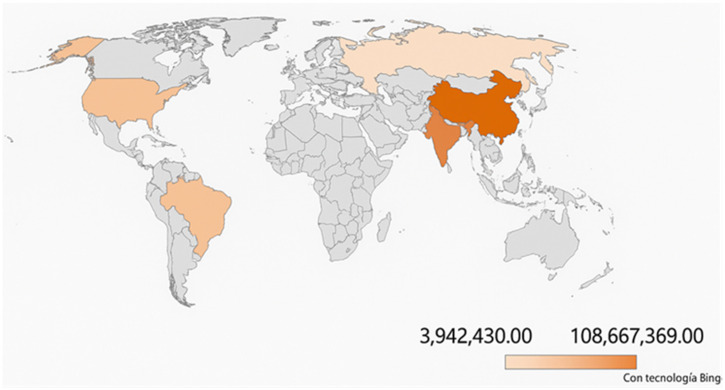
Top global countries producing household food waste in 2022 (tons/year).

Considering literature studies on agri-food waste (FW), a key concern is the substantial amount of food, still nutritionally valuable, that ends up as waste, as well as the decline in food quality (both nutritional and organoleptic) throughout the entire production chain—from field to fork [19]. Globally, about one-third of all food produced for human consumption is either lost or wasted [20]. According to the United Nations Environment Food Waste Report 2024 Programme [3], over 1.05 billion tons of food were wasted globally in 2022; of that, about 59.2 million is at the European level, mainly allocated to household consumption (for 54% of total wasted food), food manufacturing and processing (19%), restaurants and food services (11%), retail and distribution (8%), and primary production (8%). Considering the scale of global food waste, China and India are at the top of the list, wasting 108.7 million tons and 78.1 million tons annually, respectively (Figure 1). In addition, food waste has major global consequences, including USD 1 trillion in losses, 783 million people facing hunger, 8–10% of global greenhouse gas emissions, and the consumption of nearly 30% of the world’s agricultural land.

**Figure 2:**
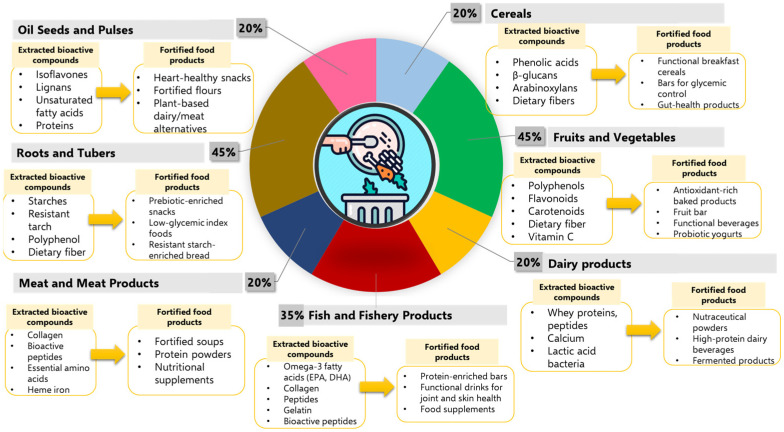
Percentage of food waste for food categories (in 2022).

The amount of FW significantly varies depending on the food category, primarily influenced by food perishability, handling practices, and storage infrastructure [21]. Fruits and vegetables account for the largest share of food waste, representing 45% of the total (Figure 2), due to their perishability and inadequate storage infrastructure. Fish and fishery products follow at 35%, being also highly susceptible to spoilage from inadequate cold chains and logistics [22]. In contrast, cereals, dairy products, meat and meat products, and oil seeds and pulses each represent a lower waste share of 20%, due to longer shelf lives.

In response to the persistent issue of food waste, the EU has set legally binding targets to reduce food waste by 2030: a 10% reduction in food manufacturing and processing and a −30% per capita reduction in retail, restaurants, food services, and households’ consumption [23].

Moreover, valorization strategies, such as the development of functional foods, nutraceuticals, and dietary supplements, represent possible opportunities to recover and valorize the nutrient-rich composition of by-products [24].

Each food category is connected to a set of nutritionally relevant compounds that can be recovered from its by-products, ranging from polyphenols, flavonoids, and vitamins in fruits and vegetables, to omega-3 fatty acids and collagen from fishery waste, and proteins, peptides, and dietary fibers in cereals and legumes (Figure 2). In these regards, it is worth highlighting the dual opportunity inherent in food waste management: reducing environmental burden while enhancing human health and nutrition.

These compounds offer nutritional and health benefits and can be effectively incorporated into a wide range of food products. Examples include baked goods (bread, cookies, cakes, muffins) [25,26], dairy products such as probiotic yogurt and fermented milk [27], meat products like sausages, pork burgers, and meatballs, cereal and fruit bars [28], edible films and coatings [29], and even beverages like juice [30].

Moreover, food by-products serve as rich sources of bioactive precursors involved in essential biochemical pathways. For example, polyunsaturated fatty acids (PUFAs), such as omega-3 and omega-6 fatty acids, are synthesized through enzymatic desaturation steps, starting from lipid intermediates. These essential fatty acids, often recovered from food waste like fishery by-products and fruit seeds, play vital roles in several biological processes [2,16,31]. 

**Figure 3:**
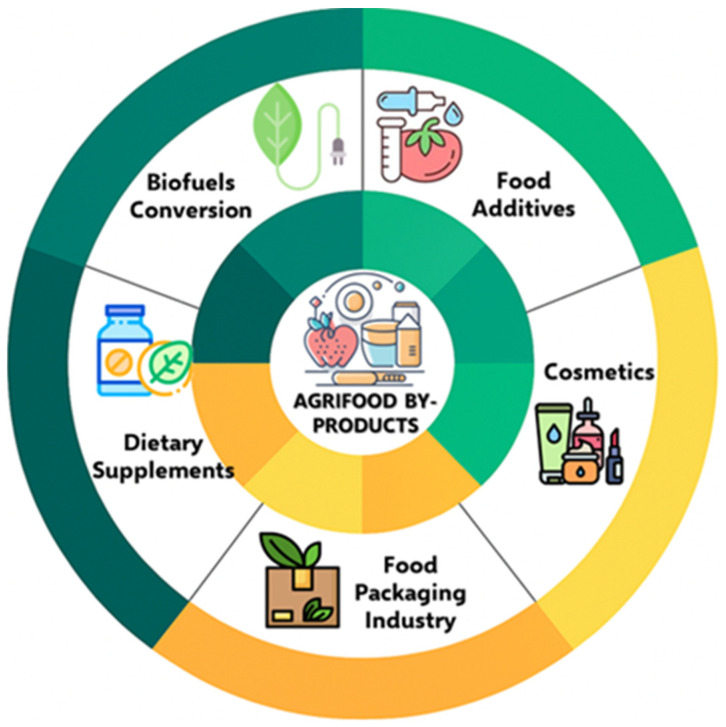
Potential agri-food by-products\applications.

Therefore, it is important to address waste management and resource depletion by efficiently using these by-products as raw materials, supporting green and sustainable technological approaches (Figure 3). Basic processes such as drying and fine grinding can significantly improve the extraction efficiency of both water- and fat-soluble bioactive compounds. Microencapsulation further enhances preservation by enclosing solid, liquid, or gaseous substances within a continuous film coating, forming capsules ranging from micrometers to millimeters in size [32].

Agri-food by-products from plant and animal industries, particularly from fisheries and meat sectors—which account for 55–65% of total by-products generated—also offer strong potential for cosmetic applications. Materials such as fish heads, skin, bones, and scales provide bioactive peptides, collagen, gelatin, oils, and calcium phosphates, that are used to produce ingredients like collagen hydrolysates, keratin, and fatty acids for cosmetic formulations [26].

The valorization of food waste can significantly enhance sustainability across its economic, environmental, and social dimensions, increasing food production efficiency and optimizing the use of existing resources to meet the demands of a growing global population that is expected to reach 9.1 billion by 2050, thus leading to an estimated +47% increase in food demand [33]. In these regards, mitigation solutions for food waste are prompted by the United Nations Sustainable Development Goals (SDGs), particularly SDG 2 (Zero Hunger), SDG 3 (Good Health and Well-Being), SDG 12 (Responsible Consumption and Production), and SDG 13 (Climate Action). In detail, food-system-related objectives mainly concern actions and mitigation solutions for (i) food security and nutrition, (ii) multifunctional agriculture and agroecology, (iii) water efficiency use, (iv) sustainable agricultural practices for stakeholders, and (v) sustainable food production and supply to promote healthier diets [22,23,24].

Table 1 maps the progress of different country groups toward SDGs, especially those most relevant to agri-food by-product valorization (e.g., SDGs 2, 3, 12, 13), based on the 2024 SDG Index Score, calculated according to Sachs et al., 2025 [34]. In detail, it is possible to note that no region has fully achieved sustainability in all relevant SDGs, but progress is visible. The global average SDG Index Score (66.30) reflects moderate progress, with most countries showing stagnation (→) or moderate improvement (➚). Countries with stronger governance and infrastructure (like OECD members and Italy) are leading in areas critical for agri-food by-product valorization, such as clean energy, innovation, and responsible consumption. Meanwhile, emerging economies show potential but face greater development and implementation challenges.

## 3. Novel Sustainable Extraction Processes/Methods for Agri-Food Bio-Products and Chemical Characterization of Bioactive Components

The current effort to recycle and valorize agri-food bio-residues, as valuable sources of raw/natural materials rich in beneficial phytochemicals, supports the principles of the green and “circular economy. These bioactive components may find significant applications as ingredients in functional food, pharmaceuticals, and cosmetic formulations.

In the last few years, traditional extraction techniques that require time-consuming treatments with solvents, namely maceration, solid–liquid and Soxhlet extraction, and hydrodistillation, have progressively been replaced by innovative approaches to optimize recovery yields, minimize environmental impact, and reduce energy consumption, at the laboratory as well as the industrial scale [35].

These novel methods include non-thermal extraction (NTE) technologies, such as ultrasound-assisted extraction (UAE), microwave-assisted extraction (MAE), supercritical fluid extraction (SFE), pressurized liquid extraction (HPLE), subcritical water extraction (SWE), high-hydrostatic-pressure extraction (HHPE), pulsed electric field extraction (PEF), and high-voltage electrical discharge extraction (HVED). For the drafting of each specific subsection—where individual extraction techniques are discussed in more detail—a bibliographic search was performed. This research focused on original articles—excluding reviews—published in the last ten years, using bibliographic databases such as Scopus, Web of Science, Google Scholar, and PubMed; only peer-reviewed articles in English were considered. The search utilized Boolean operators to combine keywords related to each extraction technique and “green extraction”, “sustainable extraction”, “non-thermal technologies”, “agro-industrial by-products”, “bio-waste valorization”, “plant matrices”, “bioactive compounds”, “polyphenols”, “terpenes”, “flavonoids”, “phenolic acids”, “functional ingredients”, and “chemical characterization”. The selection process involved screening titles, abstracts, and keywords to assess relevance, particularly for studies on high-level plant-based waste matrices like fruit and vegetable peels, pomace, and seeds, aiming to identify effective extraction strategies. Selected articles provided detailed extraction protocols (operating conditions, solvents, yields) and analytical methods (HPLC, GC-MS, LC-MS/MS, NMR). Studies comparing traditional and innovative approaches were prioritized. Table 2 summarizes the selected green extraction methods based on optimized extraction parameters for specific classes of bioactive compounds derived from agri-food by-products.

### 3.1. Ultrasound-Assisted Extraction (UAE)

Among non-thermal processes, UAE takes advantage of the mechanical and cavitation effects of the solvent to deform or disrupt the cell walls, provoke their permeabilization, and accelerate the retrieval of extractable compounds. Frequencies ranging from 20 to 100 kHz are commonly employed.

Two main sonication methods can be applied: the ultrasonic bath and the ultrasonic probe. In both cases, the raw matrix interacts with the solvent only for a few minutes, thus achieving high-purity products with preserved biological properties and reducing the consumption of organic solvents. Interestingly, extractions based on water or hydroethanolic mixtures are remarkably efficient [72].

In comparison with the conventional maceration, UAE is a green and economically viable method for food and natural products, offering several advantages: (i) shorter extraction time; (ii) lower energy and solvent consumption; (iii) improved extract quality, safety, and yields; (iv) easy use and reduced maintenance costs. These benefits allow a reduction in solid food waste and environmental pollution. However, a few drawbacks include the generation of noise (about 65 dB) by the ultrasonic machine and the need for tailored optimization of extraction parameters.

Moreover, the low extraction temperature, typically below 60 °C, helps to avoid the degradation of thermolabile components and reduce energy consumption.

Several classes of bioactive components from fruits and plants, such as antioxidants, pigments, pectin, aromas, mineral and organic compounds, have been extracted by applying UAE to many solid wastes or by-products.

For example, detailed profiles of polyphenols, instead of simply a total polyphenol content, were effectively obtained from tomato and apple peels [73], cherry by-products [74], Mediterranean fruits (apple, peach, cucumber) [75], potato peels, and olive leaves [76]; carotenoids were obtained from pomegranate peel [32] and bael fruit waste [36]; fatty acids were obtained from cocoa waste flours and soybean residue; pigments were obtained from red grape pomace; and anthocyanins were obtained from wine lees [77]. Notably, the UAE in a hydroalcoholic solvent (80% ethanol) yielded flavonoid-rich extracts from potato peel with remarkable antibacterial activity [77].

Accurate separation and quantification were achieved by advanced analytical techniques including liquid and gas chromatography (HPLC and GC) coupled with detection methods like UV-Vis, fluorescence, mass spectrometry (MS), high-resolution NMR spectroscopy, and mass spectrometry (LTQ-Orbitrap and FT-ICR) [78,79].

All these methods ensure reliable identification and characterization of recovered bioactive components and potential toxic molecules (pesticides, mycotoxins, alkaloids, and metals) [80].

### 3.2. Microwave-Assisted Extraction (MAE)

MAE is an innovative extraction technique that harnesses the electromagnetic energy of microwaves (300 MHz to 300 GHz frequency or 1 cm to 1 m wavelengths) to facilitate the solubilization and release of bioactive compounds from solid matrices [81]. The basic principle is based on the direct interaction of microwaves with polar molecules present in the solvent and matrix, generating rapid and selective heating [82,83,84,85]. During the irradiation process, the microwave radiation penetrates the solvent without substantial absorption, allowing a rapid heating of intracellular moisture, which causes the rupture of cell walls and the release of the desired compounds into the solvent [83]. At the operational level, MAE extraction can be performed in two distinct modes: pressurized microwave-assisted extraction (PMAE) and focused microwave-assisted extraction (FMAE) [83]. PMAE is conducted within a closed system that allows for precise control of pressure and temperature [86]. This method enhances solubility and solvent diffusion, leading to higher extraction yields and shorter extraction times. FMAE is performed in an open container, at atmospheric pressure [87]. The focused energy of microwaves heats the sample, leading to rapid release of volatile compounds without high-pressure conditions. Overall, MAE is particularly effective for extracting polyphenols, flavonoids, terpenes, alkaloids, and other secondary metabolites [88,89]. Accordingly, MAE emerges as an efficient technology for the production of concentrated extracts in significantly shorter times, minimizing solvent and energy consumption, and also for recovering bioactive compounds from food waste [40,41,42].

An application of MAE has involved date seeds, an abundant by-product of industrial fruit processing that is often unexploited [38]. These seeds represent a promising bio-resource rich in polyphenols and natural antioxidants. Using this method, ideal process conditions were identified as follows: temperature of about 62 °C, extraction time of 27 min, and 46% hydroalcoholic solution in ethanol (*v*/*v*). These conditions allowed an extract with a high total phenol content and marked antioxidant activity to be obtained. Indeed, HPLC-DAD allowed the identification and quantification of polyphenols such as procyanidin B1, catechin, epicatechin, quercetin-3,5′-di-O-glucoside, procyanidin B2, and syringic acid within the obtained extract.

Solaberrieta et al. [39] applied this approach to tomato seeds, a by-product of the canning industry often considered waste. In this study, MAE produced higher values of both total phenolic content (TPC) and antioxidant activity than UAE extraction. In particular, chlorogenic acid, rutin, and naringenin were found to be higher in the MAE extracts analyzed by HPLC-DAD-MS. MAE was also applied to waste from grape juice production, demonstrating the ability to extract anthocyanins from grape juice waste at different microwave powers, exposure times, and solvent/solid ratios [40]. The optimum values for the process conditions were 435 W, an exposure time of 2.31 min, and a solvent/solid ratio of 19.22 mL/g. The yield of the extract was obtained by a spectrophotometer (UV-Vis) using total monomeric anthocyanin analysis (TMA).

Likewise, to extract anthocyanins, MAE was exploited using NADES as a solvent in a study on blueberry by-products [41]. The optimized extraction conditions (T = 60 °C; heating time 2 min; matrix/solvent ratio of 20 mL/g) resulted in a TAC of 634.71 mg/100 g extract.

Another study was conducted on chestnut shells, an abundant by-product of industrial fruit processing, in Portugal, one of the largest producers of chestnuts worldwide [42]. This study reported the extraction of tannins from chestnut shells using SLE, PLE, UAE, and MAE. After a preliminary comparative evaluation, MAE emerged as the most promising in terms of extraction yield, polyphenol content, and speed of execution. The conditions for maximizing the process efficiency involved an extraction time of 5 min, a solvent/solid ratio of 50 mL/g, and a temperature of 107 °C, which achieved an extraction yield of 25 ± 3% and a total phenolic content of 344 ± 27 mg GAE/g (gallic acid equivalents) of dry extract. Furthermore, analysis of the condensed tannin content revealed a concentration of 1296 ± 68 mg catechin equivalent (CE) per gram, confirming the high quality of the extracted material. A further comparison in terms of quantitative and qualitative yield has shown that the best results for phenolic content were obtained at 93 °C with a water/solid ratio of 45 mL/g.

Pistachio shells represent a promising source of polyphenols [43]. From these by-products, a promising ethanolic MAE extract was obtained. Subsequently, the extract was fractionated by column chromatography, yielding six fractions analyzed by HPLC/ESI-MS/MS and ^1^H-NMR to identify the main antioxidant constituents.

### 3.3. Hot Pressurized Liquid Extraction (HPLE) and Subcritical Water Extraction (SWE)

Among environmentally friendly methods developed to recover bioactive compounds from food waste, HPLE, also known as ASE (accelerated solvent extraction), and SWE have recently shown great promise [90]. By solubilizing solid matrices at temperatures above the solvents’ boiling point (SWE uses water as the extraction solvent), low solvent consumption and reduced extraction time are required. The use of high pressure, which maintains the solvent in a liquid state, and the high temperature ensure matrix cell membranes break, improving mass transfer rates and facilitating high-yield extraction [91]. Both high pressure and temperature significantly influence the dielectric constant and polarity of the solvent, as is the case with water, which can extract a broad spectrum of (non)polar compounds. In particular, SWE utilizes water at approximately 200 °C (above the boiling point but below the critical temperature of 374 °C) and pressures above the critical threshold (>21.8 MPa). Compared to traditional methods, HPLE and SWE can be automated, improving repeatability and quality control [77,92].

Several food wastes and related by-products have been subjected to HPLE and SWE, recovering sterols, phenolic compounds, carotenoids, and aromatic aglycones [93], demonstrating their extraction capability and success [90] (Table 2). For example, from the residue of pineapple juice processing, F. de Andrade Maia and L.H. Fasolin have obtained high-yield bioactive phenolics, flavonoids, and carotenoids [49]. J.G. Figueroa et al. have reported the extraction optimization of phenolic compounds in avocado peel [50], whereas Katsinas, A. et al. have recovered phenolics, such as hydroxytyrosol, tyrosol, and oleuropein [51].

Moreover, Jiménez-Moreno et al. [91] have described the extraction of bioactive compounds from different agri-food by-products (Table 2): for instance, from artichoke and cardoon wastes using water as a solvent at 75 °C to recover inulin; from asparagus wastes and by-products to recover flavonols, aglycones (kaempferol, quercetin, isorhamnetin) and their glycosides, phenylpropanoids, and quinic acid; from pomegranate wastes and by-products to recover TPC, total flavonoids, and condensed and hydrolysable tannins (Table 2).

Lachos-Perez et al. used SWE to extract nonpolar flavonoids by varying the temperature-dependent dielectric constant [52], thus recovering flavanones, such as hesperidin and narirutin, from defatted orange peel. Nieto et al. achieved optimal extraction conditions of the PLE process, retrieving several polyphenols from grape stems [44]. Pereira et al. obtained extracts rich in monomeric anthocyanins and total phenolic compounds from grape marc by PLE [45]. Liew et al. optimized low-methoxyl (LM) pectin extraction using SWE with 95% ethanol from Pomelo (*Citrus grandis* (L.) Osbeck) peels [94].

Nevertheless, organic solvents used in HPLE, i.e., ethanol and methanol, even if in minimal amounts, are increasingly being abandoned, prompting the adoption of more sustainable, less toxic, and eco-friendly solvents. Examples of these alternatives are neoteric solvents [95], including DESs (deep eutectic solvents), which have emerged as a novel and promising class of solvents. NADESs (natural deep eutectic solvents), a subclass of DESs, are formulated using natural components and offer further advantages over conventional solvents such as low toxicity, cost effectiveness, biodegradability, safety, and water miscibility [96,97,98]. The implementation of SWE with NADESs as modifiers significantly enhanced the anthocyanin recovery from grape pomace compared to the sole use of SWE (see Table 2). Loarce et al. (2020) have shown a high extraction yield of catechin and epicatechin from winery by-products using a choline chloride-containing urea solution at 30% with SWE [55].

The main disadvantages of HPLE and SWE techniques regard the following: (i) high operating temperatures, which may impact the stability of bioactive compounds; (ii) high equipment cost; (iii) possibility of processing only a minimal amount of sample; (iv) high operating pressure.

### 3.4. High-Hydrostatic-Pressure Extraction (HHPE)

HHPE is an environmentally friendly, mechanical, and non-thermal technology with low energy requirements [99]. The applied pressures typically range from 100 to 900 MPa, using a solvent (typically water) as the medium for transmitting pressure. The procedure can last a few minutes, and one relevant advantage is the preservation of thermolabile compounds, due to the low working temperature [76].

Xie et al. have demonstrated enhanced emulsifying properties of the extracted pectin from potato peels after a high-pressure process. Increased galacturonic acid content and reduced esterification degree and molecular weight were also obtained [58]. Okur et al. have optimized high-pressure-assisted extraction conditions for the recovery of phenolic compounds from olive leaves, with the oleuropein content of 2.83–18.45 mg/g DW, higher than that achieved using traditional methods [57]. Moreover, Grassino et al. found that HHPE worked appropriately in polyphenol extraction from tomato peel waste due to its high yield and speed (5 min) [56].

HHPE presents some drawbacks: (i) expensive equipment; (ii) difficulty of maintenance; (iii) high pressure needed.

### 3.5. Supercritical Fluid Extraction (SFE)

Supercritical fluid extraction (SFE) is an efficient and environmentally friendly technology that employs supercritical fluids—primarily CO_2_—to extract thermolabile and bioactive compounds from complex matrices. In its supercritical state (above 31.1 °C and 7.4 MPa), CO_2_ displays advantageous solvent properties, merging gas-like diffusivity with liquid-like density. These features enable it to effectively penetrate porous materials and solubilize nonpolar or moderately polar molecules.

SFE has been extensively utilized for valorizing agri-food residues. Selected examples of its application to various agro-industrial by-products in the last five years (2019–2024) are reported below.

Tomato skins and seeds, rich in carotenoids and tocopherols, are common by-products of industrial processing. SFE has proven effective for extracting lycopene and β-carotene. Notably, lycopene recovery reached up to 97% in samples with higher moisture content (102.7 g kg^−1^ wet weight), due to the formation of a water-in-oil emulsion that enhanced extractability, with no losses attributed to isomerization. Within the wide variability in experimental parameters, the solvent ratio emerged as particularly relevant: an average of 100 kg CO_2_ kg^−1^ dry tomato pomace has yielded the highest lycopene recovery (80%) [59]. In parallel, the optimization of tomato seed oil extraction using SFE-CO_2_ has led to a yield of 16.9 wt% under best conditions (60.2 °C, 400 bar, 64.6 g min^−1^). The oil showed a favorable fatty acid profile, predominantly unsaturated fatty acids (≈80%), and notable antioxidant activity, primarily due to high γ-tocopherol content (260.3 mg kg^−1^), followed by α-tocopherol (6.53 mg kg^−1^) [60].

Citrus peel waste is a rich source of flavonoids (including hesperidin and naringin), limonoids, and terpenoids (notably limonene and linalool). SFE has been extensively applied to this high-value matrix, with extraction yield and extract composition being highly dependent on several parameters. Trabelsi et al. [61] have identified the best SFE conditions for bitter orange (*C. aurantium*) peels, i.e., 17 MPa, a static time of 53 min, and a CO_2_ flow rate of 2.9 Kg/h, thus obtaining extracts rich in fatty acid esters, terpenes, and coumarins, with isogeijerin and osthole as major components. Jerković et al. [62] have performed SFE at 10 MPa and 40 °C on *C. aurantium* and *C. sinensis* peels, yielding extracts enriched in oxygenated monoterpenes and sesquiterpenes, with limonene as the predominant compound. Notable varietal differences have been observed in minor components, including linalool, sabinene, and geranyl acetate. Recently, Mora et al. [63] have demonstrated the efficacy of SFE for extracting coumarins and polymethoxyflavonoids from the peels of *C. limonia*, *C. deliciosa*, *C. latifolia*, and *C. sinensis*. LC-MS and GC-MS analyses have revealed the presence of nobiletin, sinensetin, tangeretin, and citropten, with species-specific major compounds such as 5,6,7,4′-tetramethoxyflavanone in *C. sinensis* and nobiletin in *C. deliciosa*. Also, Romano et al. [64] have studied orange, tangerine, and lemon peels using both liquid and supercritical CO_2_, reporting that under supercritical conditions (30 MPa, 60 °C) and with 20% ethanol as co-solvent, the extraction yields as well as flavonoid contents have been maximized. Specifically, supercritical extracts were rich in flavonoids (6285.82–7828.38 μg/g dry matter, DM) and limonene (43.84–52.48%), mostly contained in orange peel extracts.

Moreover, citrus peel waste represents a valuable source of hesperidin dihydrochalcone, a sweetener additive used in the food industry for its intense flavor. Recent studies have demonstrated the feasibility of recovering hesperidin from orange peel waste through SFE. In particular, Ortiz-Sanchez et al. [65] have optimized extraction conditions at 8 MPa, 25 °C, and 10% ethanol as co-solvent, achieving a hesperidin concentration of 11.5 ± 0.03 g/kg in the extract, a 30-fold increase compared to conventional ethanol extraction.

### 3.6. Pulsed Electric Field Extraction (PEF) and High-Voltage Electrical Discharge Extraction (HVED)

Most of the conventional extraction processes are based on high mechanical stress and temperature to increase the recovery yield. However, these factors can lead to the degradation of some metabolites and prevent the selective extraction of desired compounds.

In this context, non-thermal approaches, namely pulsed electric field extraction (PEF) and high-voltage electrical discharge extraction (HVED) have emerged as promising alternatives.

PEF technology is based on the use of brief (nanoseconds to milliseconds), high-intensity electrical pulses that induce transient electro-permeabilization of cell membranes, resulting in the formation of irreversible pores in cell membranes that enhance their permeability [100]. This condition promotes a higher release of intracellular molecules and thus a higher extraction yield.

HVED applies high electrical voltages between electrodes submerged in the extraction medium, creating plasma channels that produce strong shock waves, cavitation bubbles, and UV radiation that increase the extraction capacity [101].

PEF and HVED have allowed a growing application of these protocols in food and natural fields (ultrasound, pulsed electric field, and high-voltage electrical discharge extraction of cellulose and lignin from walnut shells), including the recovery of bioactive molecules from food wastes.

#### 3.6.1. PEF Application

Luengo et al. have applied the PEF approach to recover carotenoids from tomato peel and pulp waste using a hexane/acetone/ethanol (50/25/25) mixture [66]. This study has highlighted that tomato peel is sensitive to the application of electric pulses since the use of increasing field strengths, 3 and 5 kV/cm for 90 µs, has caused a significant increase in carotenoid extraction yields. Notably, tomato pulp was not affected by electric pulse application.

Similarly, PEF applied on tomato peel and seeds for carotenoid extraction using acetone [67] and different electric field strengths (1, 2, 3, 4, and 5 kV/cm) has demonstrated an intensity-related increase in extracted carotenoids (from 35.9% to 56.4% compared to control) and polyphenols (2 kV/cm for 700 pulses).

PEF has been largely applied to grape pomace, the main by-product of wine production and a potential source of polyphenols, showing promising results in increasing the recovery of bioactive molecules [102]. In the study of Brianceau et al., several electric fields (1.2, 1.8, and 3.0 kV/cm) were applied for the ethanol/water (50:50 *v*/*v*) extraction of grape pomace [68]. The extract analysis demonstrated an increase in total polyphenols with respect to the control extraction (12.9% increase) when a 1.2 kV/cm field was used. The experimental conditions optimized by Carpentieri et al. have allowed a better permeability increase to be obtained using a 4.6 kV/cm field, with an increase in phenolic content (15%), flavonoid content (60%), anthocyanin content (23%), tannin content (42%), and antioxidant activity values (31%) [69].

#### 3.6.2. HVED Application

Spent coffee grounds (SCGs) represent a huge biomass as a residue of coffee brewing. Notably, coffee grounds are a valuable resource of molecules, including sugars, oils, and phenolics, also being a potential source of energy [103]. In this context, the HVED process has been developed to obtain a higher polyphenol extraction yield from SCGs with respect to ethanol extraction [68]. In particular, 20 min extraction time, 50 mL/g liquid-to-solid ratio, 200 mL/min flow rate, 24% ethanol concentration, and 11 kV discharge voltage have been used as the best protocol conditions, thus obtaining a polyphenol yield of 60 ± 2 mg/g, higher than solvent extraction by 20%.

Pomegranate is a fruit rich in bioactive phenolic compounds, including tannins, anthocyanins, catechins, and phenolic acids. The peel, which represents the main by-product, has a higher content of phenolics than pulp and seed and can be a good source for producing high-value antioxidants [104]. By combining different experimental conditions, namely flow rate of materials (from 8 to 12 mL/min), distance between the electrodes (from 2 to 4 mm), and liquid-to-solid ratio (from 20 to 40 mL/g) for the proper HVED approach, Xi et al. have maximized polyphenol extraction from pomegranate peel [71].

## 4. Biological Activities of Agri-Food Waste and By-Products and Mechanistic Insights

Both phytocomplexes and natural compounds derived from agri-food waste and by-products may exert pleiotropic and polyvalent mechanisms by simultaneously targeting multiple molecular pathways and compartments. These bioactivities involve multiple molecular signals such as nuclear factor kappa-B (NF-κB) and nuclear factor erythroid 2-related factor 2 (Nrf2), along with enzymes like histone deacetylases, 3-hydroxy-3-methylglutaryl coenzyme A reductase, and cyclooxygenase-2 [105,106,107].

**Figure 4:**
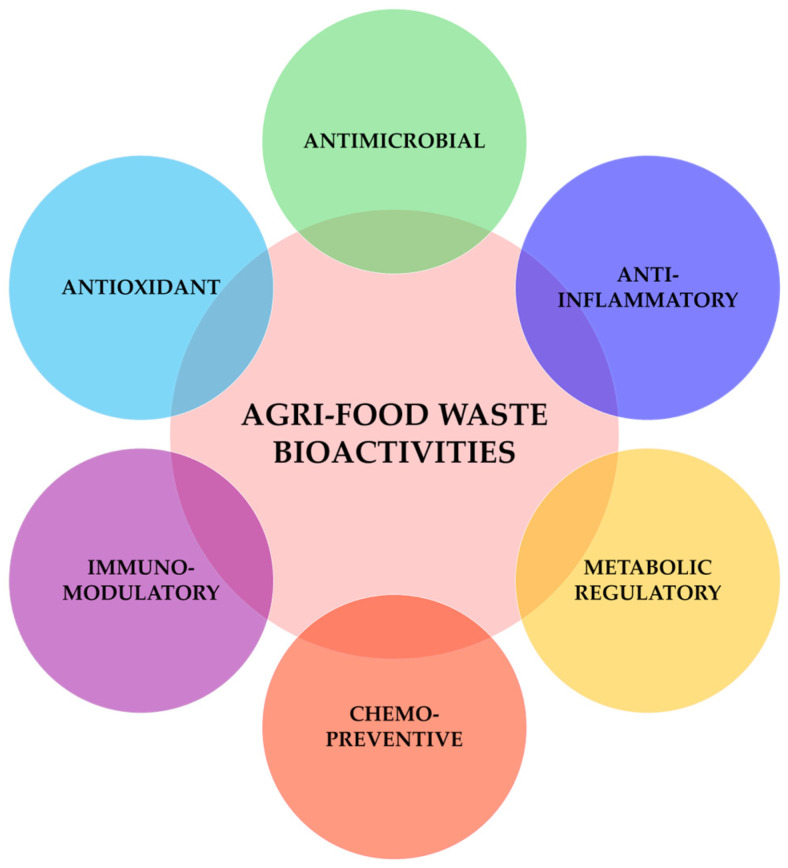
Schematic overview of the main biological activities attributed to phytocomplexes derived from agri-food waste.

The biological potential of phytocomplexes specifically derived from agri-food waste has been extensively investigated in the years, highlighting valuable activities such as antioxidant, anti-inflammatory, metabolic regulatory, antimicrobial, immunomodulatory, and chemopreventive effects (Figure 4) [95,108], some of which were also confirmed in clinical studies [109,110].

The most studied matrices include tomato, grape, apple, and pomegranate pomaces, citrus peels, cereal brans, and Brassicaceae by-products (e.g., broccoli stalks and leaves) [111,112].

The subsequent sections examine recent scientific evidence concerning the biological activities and underlying molecular mechanisms associated with phytocomplexes extracted from agri-food waste and by-products. The literature review was based on peer-reviewed articles published in the last ten years retrieved from PubMed, Scopus, and Google Scholar, using the following keywords and their Boolean combinations: “agri-food waste”, “by-products”, “bioactivities”, “phytocomplex”, “plant extracts”, “nutraceuticals”, “antioxidant”, “anti-inflammatory”, “metabolic regulatory”, “antimicrobial”, “antiviral”, “immunomodulatory”, “chemopreventive”, “molecular pathways”, “advanced glycation end-product”, “hypoglycemic”, “lipid-lowering”.

### 4.1. Antimicrobial

Agri-food by-products provide a rich source of antimicrobial and antiviral phytochemicals, including polyphenols, terpenes, alkaloids, and sulfur compounds, that act with both membrane-targeted and intracellular mechanisms. The main antimicrobial mechanisms include membrane disruption, quorum sensing interference, chelation, enzyme inhibition, and antiviral replication blockade.

Disruption of microbial cell membranes is a central antimicrobial mechanism. For instance, antibacterial and antifungal activity of essential oils is ascribed to the presence of monoterpenes, like limonene, α-terpineol, and geraniol, that increase membrane fluidity and permeability, leading to leakage of cellular content, cell lysis [113], and reduced barrier function [114].

Antibacterial activity against both Gram-positive and Gram-negative bacteria has been reported for extracts from apple pomace, grape pomace, and pomegranate peel and has been associated with the presence of lipophilic compounds, such as fatty acids, along with polyphenols, flavonoids, and phytosterols [105]. In particular, ellagic acid, the main bioactive compound in pomegranate peel, acts through several mechanisms: it disrupts bacterial cell structure, it inhibits biofilm formation, and it can synergize with antibiotics. In this respect, it has been reported that β-lactamase enzymes form stable complexes, reducing bacterial resistance to β-lactam antibiotics [114]. Ellagic acid also demonstrated promising antiviral activity against a broad range of viruses, including human immunodeficiency virus (HIV), hepatitis C virus, herpes simplex virus, and Zika virus [114]. Its mechanism of action involves the inhibition of viral entry, suppression of viral replication, and reduction in host cell susceptibility to infection.

Antiviral activity against HSV and norovirus has also been documented for other agri-food wastes, such as grape seeds and apple pomace extracts, while citrus peel extracts, containing limonene, naringin, and hesperetin, were effective against influenza virus and coronaviruses [105].

### 4.2. Antioxidant Activity

Several agri-food by-products have exhibited antioxidant properties, suggesting a promising interest for mitigating oxidative-stress-related conditions.

**Figure 5:**
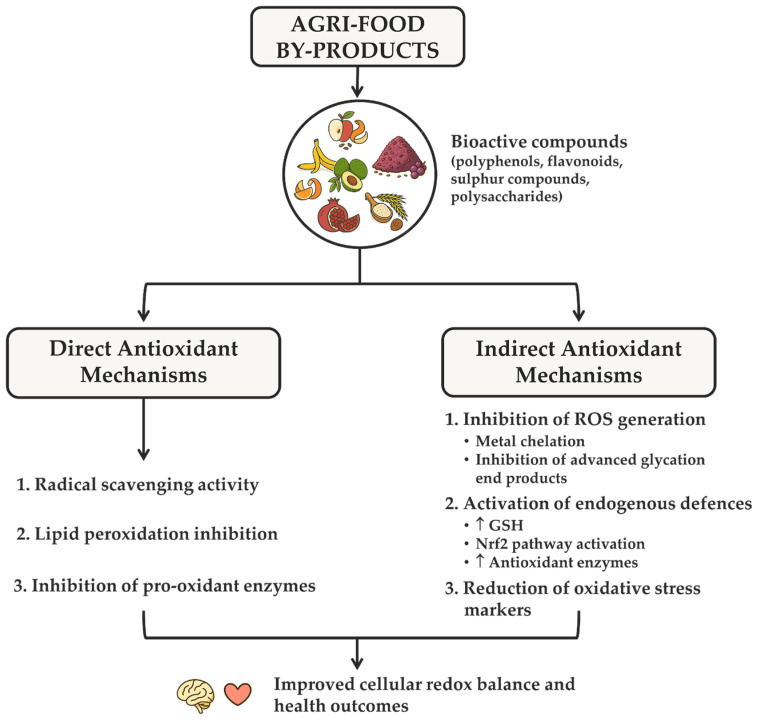
Schematic representation of the antioxidant mechanisms exerted by agri-food by-products. Bioactive compounds such as polyphenols, flavonoids, sulfur compounds, and polysaccharides contribute to redox homeostasis through both direct (e.g., radical scavenging, lipid peroxidation inhibition, inhibition of pro-oxidant enzymes) and indirect (e.g., inhibition of ROS generation, activation of endogenous antioxidant defenses via GSH and Nrf2, and reduction in oxidative stress markers) pathways, ultimately supporting improved cellular redox balance and health outcomes.

Their biological effects were ascribed to both direct antioxidant mechanisms, namely radical scavenging and lipid peroxidation inhibition, and indirect ones (Figure 5). These include the inhibition of reactive oxygen species (ROS) generation through chelating, antiglycative and reducing effects, promotion of endogenous antioxidant defenses such as antioxidant enzymes (e.g., superoxide dismutase and catalase) through Nrf2 pathway activation, and a reduction in oxidative stress markers [95,108].

Indirect antioxidant mechanisms include metal chelation, which reduces the availability of iron and copper, key catalysts in the Fenton and Haber–Weiss radical-generating reactions [115]. Phenolic-rich extracts from pomegranate peel and olive mill waste, for instance, contain compounds such as tannins, flavonoids, and phenolic acids exhibiting this kind of bioactivity [116]. Similarly, the inhibition of advanced glycation end-product (AGE) formation may further lower ROS generation [117]. Extracts from pomegranate peel, grape and apple pomace, rice husk, and coffee silverskin have demonstrated promising antiglycative activity, attributed to their composition in tannins, phenolic acids, and flavonoids, such as rutin and phlorizin [118,119].

Nrf2 is a key regulator of cellular redox homeostasis and detoxification. Many natural compounds and agri-food waste phytocomplexes activate this factor. For instance, olive pomace extracts, enriched in hydroxytyrosol and tyrosol, as well as apple and grape pomace, were found to be able to induce Nrf2 activation [120]. Similarly, ellagitannins (punicalagins and ellagic acid) and sulfur compounds (sulforaphane), abundant in pomegranate peel and broccoli by-products, respectively, promote Nrf2 activation [121].

### 4.3. Anti-Inflammatory

Extracts derived from agri-food waste exhibit direct anti-inflammatory effects by targeting multiple intracellular signaling cascades that govern the transcription of key pro-inflammatory mediators, namely tumor necrosis factor-alpha (TNF-α), interleukins IL-1β and IL-6, cyclooxygenase-2 (COX-2), and inducible nitric oxide synthase (iNOS), centrally involved in both acute and chronic inflammatory responses [105,122].

These anti-inflammatory properties have been mainly associated with the presence of polyphenols, such as flavonoids, ellagitannins (e.g., punicalagin), gallic acid, and flavonols, sulfur compounds, and terpenoids in the waste matrices [123,124].

The main involved pathways are nuclear factor kappa B (NF-κB), mitogen-activated protein kinases (MAPKs), and Janus kinase/signal transducer and activator of transcription (JAK/STAT) pathways [125].

Notably, extracts from tomato skin, pomegranate peel, grape pomace, and citrus waste have demonstrated the ability to downregulate the activation of NF-κB and inhibit cytokine release. Pomegranate peel extracts have also downregulated MAPK signaling via inhibiting the ERK1/2 and p38 phosphorylation, while grape pomace inhibited STAT3 activation [126]. Additionally, fibers from agri-food waste may control inflammation through the SCFA (short-chain fatty acid)-mediated epigenetic modulation of histone deacetylases (HDACs) and the activation of G-protein-coupled receptors.

### 4.4. Immunomodulatory

Extracts from agri-food by-products, especially cereal brans and fruit pomace, have shown the capacity to modulate the immune system and cytokine secretion [127,128]. Dietary polyphenols, such as resveratrol, catechins, and phenolic acids, are able to modulate immune responses by influencing both innate and adaptive immunity. Furthermore, dietary fibers (e.g., β-glucans, arabinoxylans, fructans) and their fermentation products (notably short-chain fatty acids) contribute to systemic immunomodulation [127,128]. Cereal bran fibers have been reported to influence immunity indirectly through the modulation of the gut microbiota and the enhanced production of short-chain fatty acids, such as butyrate, acetate, and propionate [127]. These compounds modulate immune responses by activating G-protein-coupled receptors (e.g., GPR41, GPR43), inhibiting histone deacetylases, enhancing regulatory T-cell differentiation, and reinforcing intestinal barrier integrity [129]. Moreover, cereal bran polyphenolic compounds may also contribute to the immunomodulatory effects of these fibers.

### 4.5. Chemopreventive

Agri-food by-products offer a rich source of chemopreventive agents that act with different molecular mechanisms involving antioxidant activity, inflammation control, and regulation of proliferation and apoptosis [130,131,132]. For instance, hydroalcoholic extracts of paddy waste (both husk and straw) exhibited chemopreventive effects in prostate cancer cells, through the modulation of cell cycle regulators, namely Ki-67 proliferative marker and PCNA proliferating cell nuclear antigen. Moreover, extracts from olive by-products exhibited antiproliferative, proapoptotic, and antimetastatic effects in cancer cell lines, such as colon, lung, prostate, and breast cancer cells [130]. Anticancer effects of olive mill wastewater have also been confirmed in vivo [133].

Antiproliferative properties in cancer cell models have also been reported for extracts from several waste matrices, such as pomegranate peel, tomato pomace, and citrus peels [113,131,132]. Protective effects against DNA damage induced by different toxicants have also been reported for extracts from celery waste (e.g., petioles) [134]. Further studies are needed to confirm the clinical chemopreventive role of agri-food by-products.

### 4.6. Metabolic Regulatory

Phytocomplexes derived from agri-food waste exhibited marked metabolic regulatory activities through multifactorial mechanisms.

For instance, extracts from pepper peels exhibited inhibitory effects on digestive enzymes, such as α-amylase and α-glucosidase [135], causing delayed glucose absorption and lower postprandial glycemic spikes. For instance, citrus peel extracts containing flavanones and pectic polysaccharides markedly inhibited both enzymes (α-amylase and α-glucosidase) [136]. Moreover, these extracts have been shown to inhibit the generation of advanced glycation end-products, which may contribute to hyperglycemia and diabetes complications [118,119].

Extracts from agri-food waste have also been shown to improve insulin sensitivity. For instance, apple pomace, administered in combination with a rosemary extract, improved chronic-fructose-consumption-induced insulin resistance in rats and reduced hepatic steatosis [137]. In this respect, diverse polyphenolic compounds, namely phloretin, phloridzin, ellagic acid, and cyanidin, have exhibited insulin-sensitizing effects and could be involved in these effects [105,138]. Similarly, phenolic compounds from grape pomace, namely catechin, gallic acid, chlorogenic acid, and quercetin derivatives, have been shown to competitively inhibit α-amylase enzymes, thus reducing carbohydrate digestion. Additionally, grape pomace stimulates GLUT4 translocation and activates PPARγ and Akt signaling, enhancing glucose uptake and insulin sensitivity. This potential was also confirmed in clinical studies, highlighting its potential as a functional ingredient for metabolic health support.

Along with phenolic-based matrices, cereal brans, particularly those derived from wheat, corn, and oat processing, have demonstrated significant hypoglycemic and metabolic regulatory properties [127,139]. These effects are largely attributed to their high content of arabinoxylans and β-glucans, which exert beneficial actions via multiple mechanisms [139,140]. Clinical and animal studies have consistently reported that diets enriched with cereal brans improve insulin sensitivity, reduce postprandial glucose responses, and support better glycemic control. The main molecular mechanisms involve the inhibition of intestinal carbohydrate-hydrolyzing enzymes. Additionally, glucose transporter activity can also be altered, further attenuating the postprandial glycemic peak. Beyond their enzymatic inhibition, cereal brans improve glucose metabolism via enhancing insulin sensitivity [139].

Extracts from apple and grape pomace, pomegranate peels, and broccoli by-products also showed lipid-lowering activities [105]. These effects can be mediated by multiple mechanisms, including lowering of lipid accumulation, inhibition of pancreatic lipase, modulation of lipid metabolism-related gene expression, and reduction in lipid absorption [141,142]. For instance, grape seed extract administration has been shown to reduce serum triglycerides and LDL cholesterol, while increasing HDL levels, together with increased hepatic expression of PPARα and AMPK phosphorylation. Additionally, cereal bran fibers such as arabinoxylans and β-glucans have been shown to reduce circulating triglycerides and alter bile acid metabolism [127]. Moreover, corn-bran-derived arabinoxylans enhanced lipid oxidation and mitigated liver damage in rats subjected to high-fat diets.

Dietary fibers, mainly pectin, and phenolic compounds derived from apple pomace have been shown to influence appetite regulation by modulating gut hormone secretion and microbiota composition [142,143]. Moreover, short-chain fatty acids, arising from pectin fermentation, may partially contribute to appetite regulation and energy homeostasis. However, further studies are needed to better understand the role of dietary fibers in energy intake regulation [144,145].

Table 3 summarizes the biological activities previously discussed for each considered food by-product.

## 5. Application of Bioactive Compounds Extracted from Agri-Food Waste/By-Products in Novel Functional Food Formulation, in the Pharmaceutical and Cosmetic Sectors

### 5.1. Valorization of Plant-Based Agri-Food By-Products for Novel Functional Food Development

A literature search was conducted to identify relevant scientific studies, published in the last ten years, in English, on the application of recovered bioactive compounds from agri-food waste/by-products for novel functional food development. Scientific databases searched included PubMed, Scopus, Web of Science, and Google Scholar. The search was carried out using referring to original research articles and reviews. The following keywords and their combinations were used: “agri-food by-products”, “agri-food waste”, “agri-food waste valorization”, “agri-food by-products valorization”, “novel food”, “functional food”, “innovative food”, “bioactive compounds recovery”, “agri-industrial products”. The selection process involved an initial screening of titles and abstracts, followed by a full-text review of potentially relevant articles.

While there is no single, universally accepted definition of functional foods, they are generally considered as food products that offer health benefits beyond their basic nutritional value. This category represents a rapidly expanding market, targeting consumers who are increasingly proactive in managing their own health and wellness. At the same time, diet-related conditions, such as cardiometabolic disorders including heart disease, stroke, type 2 diabetes, and obesity, remain among the most pressing global health and economic challenges, accounting for 31% of all deaths worldwide [146]. When incorporated into a balanced diet and a healthy lifestyle, functional foods offer a promising approach to preventing non-communicable diseases. Recent research confirms that incorporating fruit and vegetable by-products into products such as bread, biscuits, dairy alternatives, meat products, and drinks can improve their nutritional profile [147,148,149]. This approach supports both environmental sustainability and human health by promoting the development of functional foods that align with the principles of sustainable nutrition.

Table 4 provides an up-to-date overview of the most recent scientific contributions on the application of waste and by-products derived from agri-food, highlighting key trends such as upcycling, sustainable ingredient use, and functional food innovation. Table 5 is structured to separate in vitro or laboratory-based investigations from prototype development studies involving real food matrices or product applications.

As a sustainable alternative to conventional antioxidants, various bioactive compounds derived from food by-products have been employed to inhibit lipid oxidation in dairy products and extend shelf life. These strategies have been especially targeted at high-fat dairy items such as cheese and butter, but they have also been applied to yogurts and omega-3-enriched milk beverages, which are more susceptible to lipid degradation [150]. Research has highlighted that incorporating ingredients like grape pomace, apple peel, or tomato skin into yogurt, cheese, and fermented milk can improve antioxidant capacity, enhance gut health, and even extend shelf life through antimicrobial properties. However, challenges remain in optimizing sensory properties and ensuring product stability and consumer acceptance [150]. Notably, the inclusion of grape pomace in yogurt formulations delayed lipid oxidation and increased antioxidant capacity by up to 30%, while extending shelf life by 3–5 days, although it may affect pH and acidity [151,152]. This by-product also significantly increased phenolic and flavonoid content by 35% without compromising taste. Recently, other phenolic-rich by-product sources, such as tomato skins, broccoli stems and leaves, corn bran, and the outer leaves of artichokes, have been explored in the development of spreadable cheeses. The incorporation of broccoli stems is particularly notable, as it may enrich the product with glucosinolates, known for their positive health effects [153].

Fruit and vegetable by-products have also been added to bakery products, increasing phenolic content and antioxidant capacity [154,155]. An interesting example of by-product application as an ingredient in food products has involved the formulation of gluten-free pasta enriched with 10% or 15% tomato processing waste or linseed meal. Overall, the enriched gluten-free pasta has shown an increased content of bioactive compounds, mainly polyphenols (30%), with a strong increase in catechin, tyrosol, and protocatechuic acid. In addition, the supplementation with linseed meal provided the highest antioxidant capacity measured by ABTS (+51% at maximum level of enrichment with linseed) and FRAP assays (+126% at maximum level of enrichment with linseed), thus improving its nutritional and functional profile [156].

**Table 4:** List of recent literature articles covering the use of agri-food by-products/waste to fortify food products: from in vitro studies to prototypes.

Article Title	Authors	Year	Source/Journal	Key Topics	Reference
Section A: in vitro/laboratory studies					
High-Value Compounds in Fruit, Vegetable and Cereal By-products: An Overview of Potential Sustainable Reuse and Exploitation	Tlais, A.Z.A. et al.	2020	*Molecules*	In vitro study; analysis of bioactive compounds in food by-products, without product development	[157]
Functional Ingredients from Agri-Food Waste: Effect on Phenolic Content and Bioaccessibility in Bakery Products	Melini, V. et al.	2020	*Antioxidants*	Laboratory-based study; bakery fortification, focused on phenolics and bioaccessibility; not an effective product formulation	[26]
The use of food by-products as a novel for functional foods: Their use as ingredients and for the encapsulation process	Comunian, T.A. et al.	2021	*Trends in Food Science & Technology*	In vitro/technological approach; emphasizes encapsulation processes; functional food innovation	[24]
A Rational Definition for Functional Foods: A Perspective	Temple, N.J.	2022	*Frontiers in Nutrition*	Conceptual paper; functional food definition; health benefits; no product application	[158]
A Sustainable Waste-to-Protein System for Developing Food- and Feed-Grade Protein	Piercy & Verstraete	2022	*Green Chemistry*	Technological study on waste valorization; waste to protein; sustainable proteins; no effective food products	[159]
Application of Agri-Food By-Products in the Food Industry	Rațu R.N. et al.	2023	*Agriculture*	Narrative review; agri-food by-products; bioactive compounds; value-added foods; no product development	[155]
Innovative Foods: The Future Food Supply, Nutrition and Health	Hussain & Bekhit	2023	*Foods*	Conceptual data: novel food innovation; sustainable nutrition; alternative proteins; no experimental data or food applications	[160]
Food Waste Upcycling and Functional Foods: Innovations for Health and Sustainability	Ullagaddi, R.	2025	*African Journal of Biomedical Research*	General discussion; upcycling; food waste; functional ingredients; sustainability; not focused on food testing	[161]
Section B: application in prototypes or product development					
Food By-products as Sustainable Ingredients for Innovative and Healthy Dairy Foods	Iriondo-DeHond, M. et al.	2018	*Nutrients*	Development of dairy-based food prototypes using food by-products	[150]
Olive oil by-product as functional ingredient in bakery products. Influence of processing and evaluation of biological effects	Di Nunzio, M. et al.	2020	*Food Research International*	Functional bakery products developed and tested with olive pomace; anti-inflammatory effect	[162]
Antioxidant Properties of Gluten-Free Pasta Enriched with Vegetable By-Product	Betrouche, A. et al.	2022	*Molecules*	Gluten-free pasta prototypes enriched with vegetable by-products, experimentally tested; polyphenols; antioxidants	[156]
Can a fraction of Flour and Sugar Be Replaced with Fruit By-Product Extracts in Gluten-Free and Vegan Cookie Recipe?	Breschi, C. et al.	2024	*Molecules*	Vegan cookie formulation created and tested for nutritional properties; gluten-free	[163]
Hazelnut skin polyphenolic green extract as a promising natural antioxidant in pork burgers: Assessment of quality parameters and consumer acceptance	D’Ambra, K. et al.	2025	*Food Research International*	Prototype burgers enriched with hazelnut skin; pork burger; lipid oxidation and sensory quality evaluated	[164]
Surface application and impact of Yarrowia lipolytica grown in cheese whey as adjunct culture for innovative and fast-ripening Caciotta-like cheeses	Gottardi, D. et al.	2025	*International Journal of Food Microbiology*	Cheese whey prototype; by-product valorization; Yarrowia; fast cheese ripening; tested for microbial and textural changes	[165]

**Table 5:** List of articles covering functional foods and innovative foods derived from agri-food waste/by-products and reported by food category.

Food Category	Incorporated Agri-Food By-Products	Obtained Functional Food	Key Functional/ Nutritional Benefits	Reference
Bakery Products	Banana peel	Low-glycemic cookies. Prototype tested.	Decreased glycemic index Increased phenolic content and antioxidant activity	[166]
	Sunflower oil by-products, nut residues, cereal by-products, fruit pomace (apple, carrot, etc.)	Protein snack bars. Prototype and nutritional analysis	High protein and fiber content, enriched with antioxidants and vitamins Rich in thiamin, Ca, Mg, Zn	[167]
	Grape pomace	High-fiber muffins. Prototype tested in human crossover trial	Increased total phenolic content High protein and fiber content Decreased glycemic index Increase satiety Improved texture	[168]
	Powdered mango peel, green banana flour, pea-based powder, chickpea flour, powdered banana peel	Fiber-enriched bread Functional bread prototype developed and analyzed	– Increased dietary fiber and carotenoid content – Increased polyphenol content and antioxidant properties Increased protein, resistant starch	[154]
Dairy Products	Grape seed extract and skin flour	Plant-based yogurts. Prototypes tested for texture and antioxidant properties	Texturizing agent Enhances phenolic compound content Natural colorant Improved textural integrity and gel-forming ability	[152]
	Peer/apple stones, orange by-products, pomegranate peel, tomato peel, grape seeds, grape pomace, wine pomace, skin, and seed extract	Enhanced probiotic viability and antioxidant content	Enhanced phenolic compound content and antioxidant activity Improved probiotic viability Texturizing agent Increased antimicrobial properties	[151]
	Olive oil or fruit processing by-products	Milk alternatives. Fermented milk enriched with antioxidants	Source of protein Probiotic protection Texturizing agent Source of fiber Source of phenols Increased antioxidant capacity Colorant agent	[148]
Meat Analogs	Mosambi peel powder	Chicken meat, patties, chicken’s thigh. Meat prototype tested for antioxidant and antimicrobial activity	Enhanced antioxidant activity Antibacterial agent Increased growth of beneficial microflora	[169]
	Soymilk pulp	Okara burgers, pea patties. Texture and nutritional enhancement in meat alternatives	– Added texture and nutritional quality to plant-based meat	[170]
	Pomegranate peel, orange peel	Beef meatballs, sausages. Prototypes tested for antioxidant and antibacterial properties	Increased antioxidant capacity Antibacterial agent	[171]
Beverages	Citrus peels	Smoothies. Functional beverage with enhanced vitamin C and phenolic content	– Boosted vitamin C and total phenolic content	[172]
	Rice bran, pomegranate peel, orange pulp, and peel	Juice prototypes tested for antioxidant and lipid-lowering effects	– Lipid-lowering properties – Increased antioxidant capacity – Antibacterial agent – Enhanced juice flavor and color	[173,174]
	Grape skins	Grape-based kombucha. Probiotic kombucha with enhanced fiber and antioxidant capacity	– Major probiotic activity – Increased fiber content – Antioxidant activity	[175]

Lately, D’Ambra et al. have investigated the antioxidant potential of hazelnut skin (HS), an important by-product of the agro-industrial chain, and that of its polyphenolic extract (HSE) in relation to their impact on the quality traits of pork burgers [164]. Both HS- and HSE-enriched burgers exhibited significantly enhanced oxidative stability and color stability and reduced levels of lipid-derived volatiles, indicating a protective effect against fat oxidation. In fact, the inclusion of hazelnut skin extract in burgers led to a 25% reduction in lipid oxidation and extended product shelf life by 2–3 days. These findings underscore the potential application of hazelnut-derived antioxidant compounds as functional ingredients in meat product preservation.

Despite the abundance of studies, most of them remain limited to laboratory applications, and few address the mechanistic underpinnings of the observed bioactivities (e.g., oxidative pathway inhibition, microbiota modulation, or inflammatory cytokine regulation). Few studies explore the bioaccessibility, metabolic fate, or synergistic actions of these compounds in vivo, particularly in human subjects, limiting the translational relevance of in vitro results. Moreover, there is limited data on scalability, sensory optimization, and long-term consumer acceptance of these products. Therefore, future studies should integrate molecular mechanism analysis, in vivo and clinical validation, and standardized extraction–characterization protocols to move beyond descriptive outcomes and support evidence-based applications.

### 5.2. Valorization of Plant-Based Agri-Food By-Products for Nutraceutical Formulation

One way to provide an outlet for agri-food by-products is the formulation of functional products, namely nutraceuticals, where compounds with proven bioactive capacity are extracted and isolated to create a new product that can improve human health. The following section presents studies where new products have been developed using agri-food by-products.

Table 6 summarizes the key characteristics of these products, including the type of by-product utilized, the main bioactive compounds identified, as well as the observed functional effects, and the potential applications.

First, Lauro et al. evaluated the nanoencapsulation of orange pomace extracts to determine the stability of the formulation and its functional effect [176]. Antioxidant activity was evaluated, along with the inhibition of metalloproteases 2 and 9 (MMP-2 and MMP-9), related to inflammatory processes. It was observed that the encapsulation maintained the antioxidant activity [178]. Interestingly, the formulation derived from guava seeds exhibited the highest antioxidant capacity, despite containing lower total phenolic and flavonoid content compared to other parts of the fruit.

Tapal et al. have used the protein fraction of pea by-products to develop a hydrolysate with dual functionality: acting as a nutraceutical due to the presence of bioactive peptides and serving as a carrier for additional bioactive compounds [179]. In silico analysis of the identified peptides revealed potential biological activities, including antihypertensive effects via angiotensin-converting enzyme (ACE) inhibition and antioxidant properties. These findings were supported by in vitro experiments, where the hydrolysate demonstrated significantly higher DPPH radical scavenging activity compared to the control. Moreover, it retained its antioxidant potential during simulated gastrointestinal digestion.

On the other hand, Tenore et al. conducted a randomized, double-blind clinical trial in normoglycemic, normal-weight individuals to evaluate the effects of three nutraceutical formulations, derived from nectarine thinning waste, tomato peel, and olive leaves, on postprandial glycemic response, compared to a glucose solution used as control [180]. As detailed in Table 6, the primary bioactive components in each formulation were abscisic acid (nectarine), carotenoids (tomato), and oleuropein (olive). Nectarine- and tomato-based nutraceuticals lowered the glycemic peak without influencing insulin secretion, whereas the olive leaf formulation achieved glycemic reduction by enhancing insulin release. Bellumori et al. developed two formulations, unencapsulated and encapsulated, of a by-product from olive oil production (pâté) [181]. Antioxidant activity was assessed via the ABTS assay, and in vitro gastrointestinal digestion was performed. The encapsulated formulation exhibited higher antioxidant activity and greater total phenolic content compared to the free form. In addition, Schiano et al. investigated the valorization of nectarine thinning waste for the development of nutraceutical formulations [191]. The phenolic profile of the extract was characterized, and antioxidant capacity was assessed using DPPH, TEAC, and FRAP assays. Additionally, the inhibitory effects on α-amylase and the formation of advanced glycation end-products (AGEs) were evaluated. The formulations demonstrated strong antioxidant activity and AGE inhibition.

Buzzi et al. have explored the valorization of olive mill wastewater for the development of a multifunctional supplement for both oral and topical (cosmetic) applications [182]. A clinical study was carried out involving 46 healthy participants over an 8-week period, with a specific focus on skin health indicators. Notably, both the oral supplement and the topical formulation enhanced skin health indicators, with a synergistic effect being observed with the two treatments administered concurrently. Picerno et al. evaluated the functional properties of a novel oral formulation containing starch-based microparticles loaded with an almond skin extract [183]. The primary bioactive constituents identified were catechin and procyanidin B3 (see Table 6). The formulation significantly enhanced antioxidant capacity as measured by ORAC, TEAC, DPPH, and nitric oxide (NO) scavenging assays, outperforming pure catechin as a reference standard. Additionally, the formulation demonstrated superior inhibitory activity against AGEs and MMP-9 relative to a catechin standard. Although the dry extract exhibited limited bioaccessibility and was prone to oxidative degradation, encapsulation within the polymeric matrix improved its chemical stability and shelf life while preserving its antioxidant, anti-inflammatory, and antidiabetic properties. Sánchez-Quezada et al. developed a nutraceutical formulation based on an emulsion incorporating both lipid and aqueous fractions extracted from avocado seeds [184]. The antioxidant activity of the formulation was thoroughly assessed through DPPH, β-carotene bleaching, NO scavenging, TBARS (thiobarbituric acid reactive substances), and peroxide value assays. A toxicity assessment was also conducted using *Artemia salin* (shrimp) as a model, and simulated in vitro digestion was performed. Compared to the control, the emulsified formulations significantly enhanced antioxidant capacity, as evidenced by higher DPPH and NO scavenging activity, increased β-carotene retention, and reduced TBARS and peroxide formation. No toxic effects were observed in the bioassay model, and antioxidant functionality was retained throughout the simulated digestion process.

Castangia et al. developed a formulation derived from artichoke by-products, tested both as a dispersion and in the form of liposomes [185]. Antioxidant capacity was assessed using ORAC, FRAP, ABTS, and CUPRAC assays, alongside in vitro evaluation of α-amylase and α-glucosidase inhibitory activity. Simulated gastrointestinal digestion was performed, and the resulting digests were applied to human colon adenocarcinoma (Caco-2) cells for analysis of cell viability and oxidative stress. Both the dispersion and liposomal formulations demonstrated high and comparable antioxidant activity, which remained stable throughout the digestion process. Notably, nanoencapsulation in liposomes enhanced cellular antioxidant effects under induced oxidative stress, without affecting cell viability at low concentrations [186]. The observed inhibition of α-amylase and α-glucosidase further supports the formulation’s potential application as both an antioxidant and antidiabetic nutraceutical.

Maccarronello et al. optimized the extraction of bioactive compounds from hazelnut shells based on antioxidant activity evaluated via ABTS, FRAP, and DPPH assays [187].

The optimized extract was further assessed for reactive oxygen species (ROS) scavenging and for its inhibitory activity against *α*-amylase, *α*-glucosidase, and AGEs. The extract exhibited strong antioxidant activity relative to standard compounds such as gallic acid and catechin. It also showed superior inhibition of α-amylase compared to the reference drug acarbose, although its effect on α-glucosidase was limited. Additionally, the formulation inhibited AGEs formation by up to 80.2%; however, both catechin and aminoguanidine—used as positive controls—demonstrated higher levels of inhibition. Mehta et al. carried out a green ultrasound-assisted extraction of phenolic compounds from Sacha inchi by-products, aiming to develop a formulation with antimicrobial potential [188]. The resulting extract exhibited antimicrobial activity against various foodborne intestinal pathogens. Remarkably, in the case of *Staphylococcus aureus*, the antimicrobial efficacy of the formulation was comparable to that of the reference antibiotic, namely ampicillin. Ilgaz et al. developed a formulation based on proniosomal encapsulation of olive leaf powder, designed to serve as a carrier system for polyphenols, particularly oleuropein, the primary bioactive compound in olive leaves, which is prone to degradation during gastrointestinal digestion [189]. The formulation was evaluated for antioxidant capacity (DPPH assay), cytotoxicity (MTT assay), and cellular oxidative stress (ROS assay) using Caco-2 cells. The encapsulated formulation exhibited significant antioxidant activity even at low doses in cellular models; free oleuropein exerted a lower antioxidant effect than the formulation made with the by-product. Furthermore, the formulation was found to be non-cytotoxic at concentrations up to 200 μg/mL, supporting its potential use as a safe and effective delivery system. Finally, Mello et al. developed a nutraceutical formulation using nanostructured lipid carriers (NLCs) incorporating extracts from the peel of taperebá, a Brazilian fruit known for its high phenolic content [190].

The valorization of food and agricultural by-products for the development of novel nutraceutical formulations has predominantly focused on products intended for oral administration, as evidenced by the growing number of publications on the topic.

In contrast, the formulation of non-oral functional products remains relatively underexplored. In this respect, aside from the study by Buzzi et al. [182], previously discussed, Di Mauro et al. have developed a formulation based on olive mill wastewater, rich in polyphenols, particularly tyrosol and derivatives, that have been associated with ocular health benefits [177].

In addition, Grabauskaitė et al. investigated the valorization of strawberry, blackberry, and elderberry seed residues and rowanberry pomace through green extraction technologies, including supercritical fluid and pressurized liquid extraction [186]. The obtained extracts were evaluated for ultraviolet (UV) radiation absorption, leading to the development of a topical cream. Among the tested matrices, strawberry and blackberry extracts demonstrated the highest antioxidant capacities, with strawberry also exhibiting notable antimicrobial properties. Consequently, the pressurized liquid extract of strawberry was incorporated into the final formulation, showing superior UV absorption and antioxidant capacity. These findings support the potential of fruit by-product extracts as bioactive ingredients in natural cosmetics and cosmeceuticals.

In another study, Lorenzo et al. utilized zucchini flowers, typically discarded during harvesting, to develop a cosmetic formulation [192] with strong antioxidant activity, as demonstrated by DPPH, ABTS, and FRAP assays. In addition, protective effects against photoinduced damage were observed in HaCaT keratinocytes exposed to UV radiation and treated with the formulation. A subsequent clinical trial confirmed improved skin hydration and enhanced collagen production in participants, with no reported adverse effects.

Overall, despite the extensive research conducted in this area, the number of by-product-derived formulations that have reached the commercial stage remains limited. This gap can be partly attributed to the strict regulatory framework governing the commercialization of such products, particularly food supplements. Within the European Union, these are regulated by Directive 2002/46/EC. This regulation imposes stringent safety, efficacy, and labeling requirements that, while essential for consumer protection, may present significant barriers to market entry for novel formulations derived from agri-food waste. 

### 5.3. Application of Bioactive Compounds Extracted from Agri-Food Waste/By-Products in Pharmaceutical Applications for Therapeutic Use

In recent years, agri-food waste has emerged as a valuable resource for biomedical applications, driven by its biopolymer extraction capabilities and the presence of bioactive compounds suitable for pharmaceutical formulations. Utilizing by-products from fruits, vegetables, animals, and crustaceans addresses environmental concerns while offering new biomedical opportunities [193]. Bioactive compounds from agri-food waste are gaining increasing attention due to their antioxidant, antiviral, antibacterial, anti-inflammatory, and anticancer properties [194]. Polyphenols derived from grape seeds and skins have shown neuroprotective properties [195]. As proof of this, Pasinetti et al. have patented Meganatural-Az^®^, a purified polyphenolic extract from *Vitis vinifera* grape seeds that has recently completed a Phase II clinical trial (NCT02033941) to assess its ability to inhibit Aβ and tau aggregation in Alzheimer’s disease [196]. Another formulation, Bioactive Dietary Polyphenol Preparation (BDPP), combining grape seed extract and resveratrol, has also undergone a Phase I trial (NCT02502253). Despite their potential, grape seed polyphenols (e.g., resveratrol) face poor water solubility, rapid metabolism, and limited blood–brain barrier (BBB) permeability. To address these limitations, Loureiro et al. have developed solid lipid nanoparticles (SLNs) functionalized with an anti-transferrin receptor monoclonal antibody to enhance BBB uptake. These SLNs achieved high encapsulation efficiency (75–100%) for grape extracts (initial concentrations of 2–15 mg) and remained stable for over two months [197]. Neuroprotective effects have also been demonstrated by artichoke-based extracts. El-Nashar et al. have developed chitosan-coated SLNs loaded with artichoke bract extract and tested them in a mouse model of streptozotocin-induced sporadic Alzheimer’s disease. The treatment significantly improved memory and cognitive function, reduced inflammatory markers, and decreased Aβ and tau levels [198].

Agri-food waste materials have also been explored for wound healing applications [199,200]. The primary by-products of mango processing are peels and seeds, accounting for 35–60% of the fruit’s total weight [201] and being rich in polyphenols [202]. Al-Naymi et al. have prepared a polyurethane and hydroxypropyl methylcellulose nanofiber scaffold via electrospinning, incorporating a 20% *w*/*w* mango peel extract from *Mangifera indica* L. The formulation has shown enhanced antioxidant properties and antibacterial effects. Additionally, it suggests the promotion of angiogenesis and expedited wound healing [203]. Similarly, Veeruraj et al. have developed a collagen-based film loaded with either astaxanthin or gentamicin. Both astaxanthin and collagen were extracted from the outer skin waste of the squid *Doryteuthis singhalensis*. In vivo studies performed on rats demonstrated the effectiveness of the collagen scaffolds in promoting wound healing in both incision and excision models. Moreover, astaxanthin significantly boosted the antioxidant activity, further enhancing the wound healing properties of the film [204]. Pomegranate peel extract possesses antioxidant, anti-inflammatory, antibacterial, and wound-healing properties. Freitas-Ferreira developed a carbomer gel formulation with the extract at concentrations ranging from 1.25% to 3.75%. The formulation exhibited an antioxidant activity of 93.8%, higher than butylhydroxytoluene (BHT) and gallic acid solutions at equivalent concentrations [205].

Additionally, inedible portions of fruits, vegetables, crustaceans, and animals can also be used for biopolymer production suitable for hydrogel and scaffold preparation [206]. Chitin extracted from seafood waste (e.g., shrimp and lobster shells) can be deacetylated into chitosan. Shahzad et al. used chitosan from crab shells to create cefazolin-loaded nanoparticles (NPs), which were incorporated into an alginate–pectin film for wound dressing [207]. Cui et al. extracted cellulose from durian rind and used it to produce an organogel that, soaked in a yeast phenolic solution, showed a strong antimicrobial activity against *E. coli* and *S. aureus*, highlighting its potential as an eco-friendly wound dressing [208]. Seeds and peels of *Akebia trifoliata* var. *Australis* were extracted by Yu et al. to obtain pectin. By optimizing the pH, temperature, and pectin concentration, they formulated a stable colloidal dispersion of silver nanoparticles (AgNO_3_) in a pectin solution with strong antibacterial activity [209]. Chicken eggshells, a major by-product of the poultry industry, are an excellent calcium source for synthesizing hydroxyapatite (HA), making them a promising material for bone regeneration. Additionally, their protein-rich eggshell membrane (ESM) closely resembles mammalian extracellular matrix proteins, making it well-suited for tissue engineering applications [210]. Mensah et al. developed a reproducible method for extracting ESM using acetic acid and ethylenediaminetetraacetic acid. Thanks to its high transparency, ESM was proposed as a promising material for ocular wound dressings [211]. Chuysinuan et al. have developed an injectable dental hydrogel composed of sodium alginate and fibroin extracted from *Bombyx mori* cocoon, reinforced with eggshell-derived HA, designed to mimic alveolar bone [212].

Furthermore, agri-waste is widely recognized as a valuable resource for antibiotic production, with solid-state fermentation (SSF) being the most promising and cost-effective approach for large-scale synthesis [213,214]. Among agro-industrial by-products tested as fermentation substrates, peanut shells have shown the highest tetracycline yield (4.36 mg/g), followed by corncobs, cassava peels, and corn pomace [215]. Similarly, corn husk, corn cobs, and wheat bran have been used to synthesize rifamycin B, with corn husk demonstrating a 4-fold production increase [216]. Recently, El-Housseiny et al. explored the production of paromomycin from *Streptomyces rimosus* subsp. *paromomycinus* via SSF, using six agro-industrial substrates (i.e., sugarcane bagasse, corn bran, sunflower seed meal, wheat bran, barley, and corn bran + soybean meal). Among these, corn bran impregnated with aminoglycoside production media proved most effective, yielding a maximum paromomycin concentration of 0.51 mg/g of initial dry solids [217]. In another study, Kalaiyarasi et al. investigated antibiotic production by *Streptomyces* sp. *SD1* using various agro-industrial wastes as solid substrate, including pineapple peel, wheat bran, apple pomace, rice bran, tapioca powder, orange peel, and green gram husk [218].

While these studies highlight the potential of agri-food waste materials for various pharmaceutical applications and the synthesis of active ingredients, the transition from laboratory research to industrial development and clinical trials still needs to be reoriented to the use of biomass-based feedstocks [219].

### 5.4. Plant-Derived Extracellular Vesicles in Biomedical Applications

In recent years, plant-derived extracellular vesicles (PDEVs) have garnered significant attention for biomedical and nutraceutical applications, due to their inherent biocompatibility and bioactive composition, making them potential natural drug delivery systems. Unlike animal-derived vesicles (ADEVs), PDEVs offer advantages, namely reduced immunogenicity and lower production costs, highlighting their role in sustainable biotechnology. Derived from edible plants, PDEVs can contain various nutrients and bioactive compounds, including proteins, RNAs, lipids, vitamins, and secondary metabolites. Emerging clinical (NCT01668849; No. NCT01294072) and preclinical (NCT03493984) studies suggest that PDEVs exhibit numerous advantages over conventional synthetic carriers, opening novel frontiers for innovative drug delivery systems. Research on PDEVs has been primarily focused on edible plant tissues, namely citrus fruits, grapes, ginger, carrots, and tomatoes [220]. However, the recovery of PDEVs from agro-industrial waste remains largely underexplored, with only one study investigating PDEVs from olive vegetation water, a by-product of olive oil extraction [221]. This study reported the isolation of vesicles with nanometric dimensions, yielding approximately 8.5 × 10^11^ particles and 29.45 ± 4.4 µg of protein per 50 mL of olive vegetation water. Metabolomic profiling revealed a rich profile of bioactive compounds, such as phenolic acids, flavonoids, carbohydrate derivatives, amino acid metabolites, and intermediates from the citric acid cycle. Furthermore, proteomic analysis identified 19 distinct protein species, with some of them being involved in several biological activities. Moreover, lipidomic analysis corroborated a phospholipid-rich membrane composition characterized predominantly by phosphatidic acid and phosphatidylcholine with minimal contamination from non-vesicular lipid aggregates.

Despite the interest in PDEVs, challenges such as the lack of standardized isolation protocols and the influence of plant origin on vesicle composition hinder their broader applications. Additionally, critical factors, namely vesicle stability during digestion and the effects of food processing, require further investigation.

Given the abundance of plant-based by-products, recovering PDEVs from agro-food waste offers a promising avenue for sustainable health innovation. Future research should adopt multi-omics approaches to advance this field [222,223].

## 6. Agri-Food Waste/By-Product Valorization for Sustainable Bio-Based Packaging

The methodology entailed a structured search, selection, and analysis of literature on agri-food waste and by-products, focusing on their application in biodegradable packaging for both fresh and processed foods. The information sources were the scientific databases Scopus, Web of Science, and Google Scholar. Studies were selected referring to reviews and research articles written in English and published in the last ten years. The following keywords and their combinations were used: “agri-food waste”, “agro-industrial by-products”, “biopolymers”, “biodegradable packaging”, “sustainable materials”, “cellulose film”, “starch film”, “chitosan”, “proteins”, “citrus peel in packaging”, “spent coffee grounds in packaging”, “grape pomace in packaging”, “Pomegranate peel in packaging”, “Olive waste in packaging”.

### 6.1. Conventional Plastic Packaging and Bio-Based Alternatives

The extensive use of plastic in food, pharmaceutical, and cosmetic packaging is predominantly attributable to its versatility, cost-effectiveness, and exceptional functional properties, including elevated mechanical strength and barrier performance against moisture, gases, and contaminants [224]. However, petroleum-based plastics raise major environmental concerns due to their persistence, contribution to marine pollution, and reliance on non-renewable resources. To mitigate these issues, the packaging sector is increasingly exploring bio-based materials as sustainable alternatives [225].

Bio-based polymers, or biopolymers, can be grouped into three categories based on origin and production method [226].

The first includes polymers extracted directly from biomass, namely starch, cellulose, casein, and gluten. These polymers are typically obtained through mechanical, chemical, or enzymatic processes and retain the macromolecular structure of the source material. Although biodegradable and eco-friendly, they often exhibit limited mechanical and barrier properties [224].

The second group comprises polymers synthesized from renewable monomers, such as polylactic acid (PLA), a biopolyester produced by polymerizing lactic acid derived from fermentation of carbohydrate-rich agricultural feedstocks. PLA is one of the most commercially successful bioplastics due to its favorable mechanical properties and processability, enabling its use in conventional plastic manufacturing equipment.

The third category includes microbial-derived polymers, mainly polyhydroxyalkanoates (PHAs), synthesized by various microorganisms under nutrient-limited conditions. PHAs are fully biodegradable and show good mechanical and thermal properties [227].

Despite their potential, biopolymers face several challenges, primarily related to performance, processability, and costs.

These limitations have spurred growing interest in the development of biopackaging materials—sustainable packaging solutions derived from renewable biological sources such as plant starches, lignocellulosic biomass, proteins, and polysaccharides [228]. These materials can be biodegradable and compostable, and in some cases offer additional bioactive functionalities. However, their market share remains modest: bio-based plastics represent only ~1% of total plastic production in Europe [224]. Barriers include high production costs, limited barrier properties, and scalability issues.

To overcome these, strategies such as material optimization, plastic reduction through design, and inclusion of recycled content have been proposed [229].

### 6.2. Biopolymers Derived from Agro-Industrial By-Products

A promising approach is the development of bio-based packaging from renewable sources, particularly agricultural and marine by-products [230].

Polysaccharides and proteins from these residues are attractive for their biodegradability, availability, and functional properties suitable for packaging applications [167]. Chitosan, derived from the deacetylation of chitin in crustacean shells, shows antimicrobial, film-forming, and mechanical qualities suitable for food packaging. Likewise, polysaccharides like starch and cellulose from agricultural residues have been widely studied for their potential in biodegradable film production [231].

Proteins like zein and gluten from plant sources also offer potential. However, issues namely hydrophilicity, brittleness, and inconsistent mechanical behavior limit their broader use. To overcome these, strategies like polymer blending, chemical modification, and plasticizer incorporation have been explored to improve material performance [232,233].

#### 6.2.1. Polysaccharides

Polysaccharides, including cellulose, starch, and chitosan, represent a significant category of bio-based polymers used for sustainable packaging.

Cellulose, the most abundant natural polymer, is valued for its biocompatibility, non-toxicity, biodegradability, and renewability. However, pure cellulose films lack antioxidant and antimicrobial properties, which limits their food preservation function.

To enhance their functionality, active cellulose-based films have been incorporated with bioactive agents, either as blends with soluble antimicrobial or antioxidant compounds (e.g., natural extracts, polyphenols) or as composites with nano- or microparticles dispersed in the matrix [234]. These additives improve biological activity and affect physical properties like moisture sensitivity and mechanical strength [234].

Starch-based materials are widely explored for sustainable packaging. However, native starch films show poor mechanical strength, high brittleness, and moisture sensitivity, limiting food packaging use. To overcome these drawbacks, strategies such as chemical/physical modifications, blending with other biopolymers, adding plasticizers (e.g., glycerol, sorbitol), and reinforcing with nanoparticles have been applied [235,236].

In particular, nanomaterials like cellulose nanocrystals, nanoclays, or chitosan nanoparticles enhance flexibility, strength, and gas/water barrier performance while maintaining biodegradability [237].

Moreover, recent advances in starch-based hydrogels and aerogels further expand their role in active packaging, offering controlled release of bioactive compounds such as antioxidants, antimicrobial agents, or essential oils. Aerogels, thanks to their high surface area and tunable porosity, show potential as carriers in smart packaging, where controlled interactions with food or the surrounding environment are required to extend shelf life and improve safety [238].

Chitosan, a biopolymer obtained through the deacetylation of chitin from crustacean shells, is valued for its biodegradability, film-forming capacity, and intrinsic antimicrobial activity [239]. Recent research has increasingly focused on improving the performance of chitosan-based films to meet the specific demands of food packaging applications. One promising approach involves chemical crosslinking with natural compounds to enhance the film’s stability and bioactivity. In a study by Wang et al. [240], chitosan–vanillin films effectively inhibited the growth of Botrytis cinerea, the fungus responsible for gray mold in strawberries, thus prolonging the shelf life and maintaining the quality of the fruit under refrigerated storage conditions.

Additionally, the incorporation of metal or metal oxide nanoparticles has enhanced chitosan-based nanocomposites for active and intelligent packaging, enabling responsiveness to environmental changes and prolonging the shelf life of perishable foods [241].

#### 6.2.2. Proteins

Proteins from agro-industrial waste, such as gelatin, zein, and soy protein isolate, are gaining attention as sustainable and biodegradable alternatives to conventional packaging materials due to their film-forming ability, gas barrier properties, and suitability for food applications.

Gelatin, obtained through the hydrolysis of collagen found in animal by-products like skin, bones, and connective tissues, is valued for its film-forming properties but suffers from brittleness and moisture sensitivity. To improve its performance, recent studies have explored reinforcing agents and physical treatments.

For example, the addition of bacterial cellulose nanofibers has been shown to significantly improve the mechanical strength and reduce the water vapor permeability of gelatin films, as demonstrated in their application to fresh strawberries, where the modified films contributed to moisture control and preservation of visual quality during storage [242]. Additionally, exposure to ultraviolet (UV) radiation has been employed to cross-link gelatin matrices, resulting in films with increased tensile strength and reduced solubility. These advancements contribute to the development of gelatin-based films with enhanced performance suitable for food packaging applications [243].

Zein (a maize-derived prolamin) and soy proteins are widely used in biodegradable films for their film-forming abilities. Recent studies have aimed to enhance their functionality. For instance, soy-protein-isolate-based films incorporated with natural extracts such as Zanthoxylum bungeanum leaf extract have shown improved tensile strength, water resistance, and antioxidant activity [244]. 

### 6.3. Incorporation of Agro-Food Waste into Biopolymeric Matrices

The incorporation of natural additives, such as agro-industrial by-products, into bio-based packaging materials is a promising strategy aimed at enhancing their antimicrobial and antioxidant properties. This approach offers multiple advantages: it improves the functional attributes of packaging, extends the shelf life of perishable products, and supports the principles of a circular bioeconomy by valorizing waste streams. In recent years, various studies using this approach have been carried out [245]. Among the most explored additives, citrus peel, spent coffee grounds, grape pomace, pomegranate peel, and olive waste are the most promising, with each one exhibiting unique physicochemical and bioactive properties that contribute to packaging performance. A summary of the selected studies is presented in Table 7, highlighting the specific by-product employed, the type of polymer matrix used, and the observed functional effects of the resulting composite materials.

#### 6.3.1. Citrus Peel

Citrus peel by-products are promising additives for bio-based packaging due to their antioxidant and antimicrobial effects. Various studies have explored their integration into polymeric matrices to develop active and intelligent packaging solutions.

Bitter orange peel extract, encapsulated in gelatin nanoparticles via coaxial electrospray, has shown strong antioxidant and antibacterial activity against E. coli O157:H7, suggesting it can be applied in food packaging to inhibit foodborne pathogens and extend shelf life [246].

In another study, essential oils from sweet lime peels added to biodegradable films enhanced antimicrobial efficacy at 3% concentration, extended fish fillet shelf life, and improved mechanical and barrier properties [247].

On an industrial scale, PLA/PHB films with encapsulated citrus peel extracts provided controlled antioxidant release and antimicrobial effects (*S. aureus*, *E. coli*) and met food contact safety standards [248].

Furthermore, Arslan et al. [249] investigated the incorporation of fermented lemon peel extracts into chitosan-based films. The resulting films exhibited significant antifungal properties against fungi responsible for post-harvest decay. Additionally, the films demonstrated increased hydrophilicity and enhanced ultraviolet (UV) shielding effects, indicating their potential to improve the preservation of perishable food products by protecting them from fungal contamination and UV-induced degradation.

#### 6.3.2. Spent Coffee Grounds

The integration of coffee-derived by-products, particularly spent coffee grounds (SCGs), into biopolymer matrices has been explored to develop sustainable packaging materials with enhanced functional properties.

Masssijaya et al. [250] reported that the incorporation of SCGs (particularly at 5% concentration) as a filler in poly(3-hydroxybutyrate-co-3-hydroxyvalerate) (PHBV) biopolymer composites enhanced the mechanical properties and hydrophobicity of the composite films, suggesting their potential application in sustainable packaging. Mendes et al. [251] reported that the incorporation of SCGs (particularly at 5% concentration) as a filler in poly (3-hydroxybutyrate-co-3-hydroxyvalerate) (PHBV) biopolymer composites improves the barrier properties. SCG oil has also improved antioxidant activity and texture in κ-carrageenan-based edible films [252].

#### 6.3.3. Grape Pomace

The use of grape pomace, a by-product of the winemaking process, as an additive in biopolymer formulations has garnered significant attention for developing sustainable packaging materials with enhanced functional properties.

Recent work by Amiri Samani et al. [253] focused on the co-extraction of pectin and phenolic compounds from red grape pomace using ultrasound–microwave-assisted extraction. Pectin was blended with red kidney bean protein isolate to form biodegradable films with excellent barrier, thermal, and mechanical properties. The addition of grape pomace extract further enhanced the antioxidant and antimicrobial activity of the films.

Another investigation focused on blending grape pomace with bio-based poly(butylene succinate) (BioPBS) to create biocomposites. The addition of grape pomace, particularly at 40 wt%, enhanced the flexural and impact strengths of the composites. The incorporation of 3 wt% maleic anhydride-grafted BioPBS further improved interfacial adhesion, suggesting the viability of these biocomposites for packaging applications [255]. Furthermore, research by Gubitosa et al. [254] involved embedding polyphenolic extracts from grape pomace into sodium alginate-based films. These hybrid films exhibited UV-light screening and enhanced antioxidant properties.

#### 6.3.4. Pomegranate Peel

Pomegranate peel, a by-product of pomegranate juice production, has been effectively utilized as an additive in biopolymer-based packaging films, imparting notable antioxidant and antimicrobial properties [122]. In a specific application, Diaz-Herrera et al. [256] developed pectin-based edible films incorporating pomegranate peel polyphenols. These films demonstrated low water vapor permeability and significant antioxidant activity, attributed to the bioactive compounds from the peel, suggesting their efficacy in preserving food quality.

Furthermore, a recent study by Ahmad et al. [257] explored the integration of pomegranate rind extract into carboxymethyl cellulose (CMC)-based films, showing remarkable antimicrobial performance, inhibiting the growth of both Gram-positive and Gram-negative bacteria, and exhibiting superior antioxidant properties compared to control films.

#### 6.3.5. Olive Waste

In a study by Lammi et al. [258], olive pomace was fractionated into stone-rich, pulp-rich, and crude pomace fractions that were then used as fillers in polyhydroxybutyrate-co-valerate (PHBV) matrices. The incorporation of a 30% pulp-rich fraction into PHBV resulted in a 36% reduction in stress at break, indicating a moderate impact on mechanical properties and biodegradability potential. PLA films coated with olive leaf extract showed strong antimicrobial activity against *S. aureus*, supporting their applicability in active food packaging to inhibit bacterial growth [259].

Furthermore, research focused on creating active biodegradable films by incorporating phenolic extracts from olive oil mill by-products into a carboxymethylcellulose (CMC) matrix. The resulting films exhibited high antioxidant content and improved UV barrier properties, reducing oxidation in polyunsaturated fatty acid-rich oils, thus enhancing the shelf life of packaged foods [260].

In a similar approach, Apicella et al. [261] developed innovative packaging using commercial biodegradable blown film, made by poly(lactic acid) and poly(butylene adipate-co-terephthalate) blend, incorporated with polyphenolic extracts derived from olive mill wastewater. The incorporation of the olive extract within a 1–3% *w*/*w* range into the multilayer films did not significantly affect the barrier performance of the systems. Surface wettability measurements revealed a stronger affinity of the polymer matrix with the 95% ethanol solvent, which was subsequently selected as the target food simulant for the release tests. Finally, the DPPH antioxidant activity assay performed on the food simulant highlighted the potential of the produced films to serve as carriers for the controlled release of the antioxidant agent.

## 7. Strategic Research Priorities and Innovation Roadmap

The valorization of agri-food by-products has evolved from a peripheral concept to an essential pillar for developing resilient, health-oriented, and circular food systems. This review emphasizes that strategic investment in interdisciplinary research, scalable technologies, and harmonized policies is crucial to transforming waste streams into value chains.

Looking ahead, the advancement of this field requires not only continued innovation but also structured and collaborative efforts. The transition to a regenerative bioeconomy will not only depend on innovation but also a collective commitment—from scientists, policymakers, industries, and society as a whole—to redefine waste as a resource that benefits both planetary and human health.

This section outlines the key strategic priorities that should inform the field’s progression.

Future investigations should aim to elucidate the following: (1) the most bioavailable and clinically relevant classes of bioactive compounds present across various agri-food matrices; (2) the dose–response relationships, safety profiles, and synergistic effects of compound combinations; (3) the impact of food matrix interactions and formulation technologies on enhancing bioavailability and functionality.

The following priority research directions have been identified: (1) improving the bioavailability and clinical validation of recovered bioactives through robust in vivo and human studies; (2) optimizing sustainable extraction technologies for scalability and cost-efficiency; (3) mechanistic modeling of the health effects of bioactive compounds derived from agri-food by-products/waste; (4) developing standardized protocols for quality control and regulatory compliance.

To achieve these goals, it will be essential to establish interdisciplinary consortia comprising experts in different disciplines, such as food science, biotechnology, clinical nutrition, regulatory affairs, environmental sustainability, and policy experts. Additionally, the creation of shared infrastructures and data platforms to standardize methodologies, compound characterization, and efficacy assessments will be crucial. The development of certification schemes and guidelines aimed at fostering industry adoption is also necessary. These collaborative networks can facilitate knowledge exchange, accelerate innovation, and align scientific efforts with market and policy needs.

While numerous extraction technologies have demonstrated potential at the laboratory scale, the challenge of industrial scalability persists. A structured Technology Readiness Level (TRL) framework should be employed to (1) identify critical milestones necessary for upscaling processes from pilot to industrial levels; (2) develop modular, cost-effective biorefinery systems that can be tailored to regional waste streams; and (3) facilitate techno-economic and life cycle assessments to ensure the commercial and environmental sustainability of processes. The development of Technology Readiness Level (TRL) roadmaps will help stakeholders assess the maturity of valorization technologies, from lab-scale proof-of-concept to industrial implementation, thereby guiding investments and informed policy decisions (Table 8).

By embedding these strategic priorities into national and international research agendas, and by fostering coordinated action across academia, industry, and governance, the valorization of agri-food by-products can evolve from an emerging research domain into a mature and impactful component of the bioeconomy. With continued innovation, investment, policy support, and scientific collaboration, the valorization of agri-food waste/by-products can become a cornerstone of a regenerative bioeconomy that aligns with global sustainability goals, addresses public health challenges, and promotes inclusive economic growth.

## 8. Conclusions

The valorization of agri-food by-products has emerged as a key driver in the transition toward more sustainable and circular food systems. As highlighted in this review, these by-products, long considered waste, are now recognized as valuable resources rich in bioactive compounds useful in preventing or treating chronic diseases, maintaining well-being, or enhancing human health. These bioactive molecules, recovered from agri-food waste, demonstrate strong potential for applications in the food, nutraceutical, pharmaceutical, cosmetic, and packaging sectors, thereby expanding the global food market due to their abundance, low cost, and availability, while advancing the goals outlined in the United Nations’ 2030 Sustainable Development Goals (SDGs). The adoption of innovative extraction technologies—such as ultrasound-assisted, enzyme-based, and supercritical fluid methods—has improved both the efficiency and sustainability of bioactive recovery, reducing the use of harmful solvents and their associated environmental impact.

By reframing waste as a resource, the agri-food sector is positioned not only to mitigate environmental degradation and reduce its carbon footprint, but also to support public health through the development of functional foods, nutraceuticals, and dietary supplements that address the rise in chronic diseases associated with modern dietary patterns. Furthermore, the integration of bioactive compounds into biodegradable and active packaging offers an environmentally conscious alternative to petroleum-based plastics, fostering synergies between health, innovation, and sustainability.

Nevertheless, several challenges must be overcome to fully realize the potential of this valorization approach. The lack of harmonized regulatory frameworks, particularly regarding the safety, labeling, and standardization of compounds derived from food waste, remains a significant barrier to commercialization and consumer trust. Additionally, many of the extraction processes, although efficient at the laboratory scale, require further optimization to become economically viable and scalable for industrial applications.

Variability in phytochemical composition, resulting from differences in plant genotype, environmental conditions, and processing methods, necessitates robust protocols for quality control and reproducibility. Scientific efforts must also focus on improving the bioavailability, efficacy, and metabolic profiling of the recovered bioactive compounds through robust in vivo studies and clinical validation. This will be critical in substantiating health claims and enabling integration into mainstream health and nutrition strategies.

Finally, realizing the full potential of agri-food waste valorization requires more than technological advancement. It demands coordinated action among industry stakeholders, policymakers, and the scientific community to ensure the scalability, regulatory alignment, and social acceptance of these solutions.

## Figures and Tables

**Table 1:** Mapping the progress of UN member states towards achieving the SDGs most involved in agri-food by-product valorization. Data elaborated based on Sachs et al., 2025 [34]. Green: goal achievement; yellow: challenges remain; orange: significant challenges; red: major challenges; grey: insufficient data. Time series indicate the following: ↑, on track or maintaining achievement; ➚, moderately increasing; →, stagnating; ↓, decreasing.

Country	2024 SDG Index Score	SDG2: No Hunger		SDG3: Good Health and Well-Being		SDG12: Responsible Consumption and Production		SDG13: Climate Action	
Italy	79.29		**→**		**➚**		**→**		**➚**
BRICS members *	67.89		**→**		**➚**		**→**		**→**
BRICS Plus members ^†^	67.01		**→**		**➚**		**→**		**→**
East and South Asia	66.53		**→**		**➚**		**→**		**→**
Eastern Europe and Central Asia	70.56		**→**		**➚**		**→**		**→**
Latin America and the Caribbean	70.15		**→**		**→**		**➚**		**↑**
Middle East and North Africa	65.60		**↓**		**→**		**➚**		**➚**
OECD members	77.25		**→**		**➚**		**→**		**→**
Small island developing states	64.62		**→**		**→**				**→**
Sub-Saharan Africa	53.73		**→**		**→**		**↑**		**↑**
Low-income countries	51.02		**→**		**→**		**↑**		**➚**
Lower-middle-income countries	63.18		**→**		**➚**		**↓**		**→**
Upper-middle-income countries	71.04		**→**		**➚**		**→**		**→**
High-income countries	77.61		**→**		**➚**		**→**		**→**
World	66.30		**→**		**➚**		**→**		**→**

* BRICS members: Brazil, Russia, India, China, South Africa; ^†^ BRICS Plus members: Egypt, Ethiopia, Iran, Saudi Arabia, United Arab Emirates.

**Table 2:** Main green extraction methods and optimized parameters for the recovery of bioactive compounds from agri-food by-products.

Green Extraction Methods	Extraction Conditions	Agri-Food By-Products	Main Extracted Metabolites/Bioactive Metabolites	Analytical Methods	Ref.
UAE	T ^1^: 45 °C S ^2^: ethanol (50%) t ^3^: 4–10 min	Bael fruit pulp	Polyphenols, flavonoids, carotenoids	UV-Vis	[36]
	T: 20–60 °C S: soy oil t: 30 min	Pomegranate peel	Carotenoids	HPLC-DAD	[32]
	T: 0 °C S: ethanol 80% t: 27 min	Chestnut burs, shells, and leaves	Polyphenols	HPLC-DAD, LC-MS	[33]
	T: 67 °C S: water (pH 1.5) t: 28 min	Grapefruit	Pectin	FTIR, SEM	[37]
MAE	T: 62 °C S: ethanol (46%) t: 27 min	Date seeds	Polyphenols	HPLC-DAD	[38]
	T: 80 °C S: ethanol (63%) t: 15 min	Tomato seeds	TPC, polyphenols	UV-Vis, HPLC-DAD-MS	[39]
	PW ^4^: 428.02 W S: water S/M ^5^: 18.43 mL/g t: 2.23 min	Grape juice waste	TMA	UV-Vis	[40]
	T: 60 °C S: NADES (glucose/glycerol/lactic acid, 1:2:5) S/M: 20 mL/g t: 30 min	Blueberry by-products	TPC, anthocyanins	UV-Vis	[41]
	T: 107 °C S: water S/M: 50 mL/g t: 5 min	Chestnut shells	TPC, TAC	UV-Vis	[42]
	PW: 1000 W S: ethanol (50%) t: 4.5 min	Pistachio shells	TPC, TFC, polyphenols, flavonoids	UV-Vis, HPLC/ESI-MS/MS, NMR	[43]
PLE	T: 120 °C S: ethanol (30%) P ^6^: 10.3 MPa t: 10 min	Grape stems	Polyphenols	RP-HPLC-PAD-MS	[44]
	T: 40 °C (anthocyanins) T: 100 °C (phenolics) S: ethanol–water	Grape stems	anthocyanins, phenolic compounds	UHPLC-QToF-MS, UHPLC-UV-Vis	[45]
	T: 75 °C S: water P: 10 MPa t: 27 min	Artichoke and cardoon wastes	Inulin	GC-MS	[46]
	T: 65 °C S: ethanol (50%) t: 30 min	Asparagus wastes and by-products	Flavonols, phenylpropanoids	HPLC-MS	[47]
	T: 40 °C S: water P: 10.3 MPa t: 5 min	Pomegranate wastes	TPC, flavonoids, tannins	HPLC-DAD-MS	[48]
	T: 100 °C S: ethanol (50%; 75%)	Pineapple residues	Phenolics, flavonoids, carotenoids	HPLC-DAD-MS, UV–Vis	[49]
	T: 200 °C S: ethanol (50%)	Avocado peel	Phenolic compounds	HPLC-DAD-ESI-TOF-MS	[50]
	S: ethanol (different %) P: 10 MPa t: 20 min	Olive pomace	Hydroxytyrosol, tyrosol, oleuropein	HPLC-DAD- MS/MS	[51]
SWE	T: 150 °C P: 10 MPa F ^7^: 10 mL/min	Defatted orange peels	Flavanones	HPLC-UV	[52]
	T: 60 °C S: NADES 30% (*w*/*w*) of choline chloride and oxalic acid	Grape pomace	Anthocyanins	HPLC-DAD-ESI-MS/MS	[53]
	T: 120 °C S: ethanol (95%) P: 3 MPa	Pomelo (*Citrus grandis* (L.) Osbeck) peels	Low-methoxyl (LM) pectin	FT-IR	[54]
	T: 100 °C S: choline chloride with urea (30%) P: 10 MPa t: 10 min (two cycles)	Winery by-products	Catechin, epicatechin	HPLC-DAD	[55]
HHPE	T: 45 °C; 55 °C S: methanol (50%;70%) P: 600 MPa	Tomato peels	Polyphenols	HPLC-DAD	[56]
	P: 300–500 MPa t: 5–10 min	Olive leaves	TPC	HPLC-DAD, FT-IR	[57]
	P: 200 MPa t: 5 min	Potato peels	Pectin	FT-IR, NMR	[58]
SFE	T: 80 ± 1 °C P: 38 MPaS/M: 103 ± 4 Kg CO_2_ Kg^−1^ d.m.	Tomato skins and seeds	Lycopene	HPLC- UV-Vis	[59]
	T: 60.2 °C P: 40 MPa F (CO_2_): 64.6 g min^−1^	Tomato seeds	Tocoferols, fatty acids	HPLC-DAD, GC-MS, GC-FID	[60]
	S: ethanol P: 17 MPa F (CO_2_): 2.7 Kgh^−1^ t: 120 min	Bitter orange (*C. aurantium*) peels	Coumarin (osthole)	GM-MS	[61]
	T: 40 °C P: 10 MPa F (CO_2_): 1.76 Kgh^−1^	*C. aurantium* and *C. sinensis* peels	Terpenes, coumarins	GC-FID/MS	[62]
	T: 40 °C P: 8 MPa F (CO_2_): 1.2 kgh^−1^	Peels of *C. limonia*, *C. deliciosa*, *C. latifolia*, and *C. sinensis*	Coumarins, polymethoxyflavonoids	LC-MS, GC-MS	[63]
	T: 60 °C S: ethanol (20%) P: 30 MPa	Orange, tangerine, and lemon peels	TPC, polyphenols, terpenes	UV-Vis, HPLC-DAD, GC-MS	[64]
	T: 25 °C S: ethanol (10%) P: 8 MPa	Citrus peel	TPC, hesperidin	UV-Vis	[65]
PEF	S: hexane/acetone/hanol (50:25:25) E ^8^: 5 kV/cm tp ^9^: 90 µs	Tomato peels	Carotenoids	UV-Vis, HPLC/DAD	[66]
	S: acetone E: 1,2,3,4, and 5 kV/cm tp: 15 µs	Tomato peels and seeds	Carotenoids	UV-Vis, HPLC/DAD	[67]
	S: ethanol/water E: 1.2, 1.8, and 3.0 kV/cm tp: 100 µs	Grape pomace	TPC	UV-Vis, HPLC/DAD	[68]
	S: ethanol/water E: 4.6 kV/cm tp: 20 µs	Grape pomace	TPC, flavonoids, anthocyanins, tannins	UV-Vis, HPLC/DAD	[69]
HVED	S: ethanol (24%) LSR: 50 mL/g E: 11 kV/cm F: 200 mL/min t: 20 min	Spent coffee grounds	TPC	UV-Vis	[70]
	S: water S/M: 35 mL/g E: 29 kV/cm F: 12 mL/min	Pomegranate peels	Total polyphenols	UV-Vis	[71]

^1^ T: temperature, ^2^ S: solvent, ^3^ t: time, ^4^ PW: microwave power, ^5^ S/M: solvent/matrix ratio; ^6^ P: pressure, ^7^ F: flow, ^8^ E: electric field intensity, ^9^ tp: time pulse, UAE: ultrasound-assisted extraction, MAE: microwave-assisted extraction, PLE: pressurized liquid extraction, SWE: subcritical water extraction, HHPE: high-hydrostatic-pressure extraction, SFE: supercritical fluid extraction, PEF: pulsed electric field extraction, HVED: high-voltage electrical discharge extraction.

**Table 3:** Food by-products and observed biological activities.

Source	Biological Activity	Source	Biological Activity
Apple pomace	Antimicrobial [105], antioxidant [118,120], metabolic regulatory [105,137,143]	Grape seeds	Antimicrobial [105]
Broccoli by-products	Antioxidant [120], metabolic regulatory [105]	Olive by-products	Antioxidant [116], chemopreventive [130,133]
Celery by-products	Chemopreventive [134]	Pepper peel	Metabolic regulatory [135]
Cereal brans	Immunomodulatory [127], metabolic regulatory [127,139]	Pomegranate peel	Antimicrobial [105,114], antioxidant [116,118,121], anti-inflammatory [126], chemopreventive [131], metabolic regulatory [105]
Citrus peel	Antimicrobial [105], chemopreventive [113]	Rice husk	Antioxidant [118]
Coffee silverskin	Antioxidant [118]	Tomato pomace	Chemopreventive [132]
Grape pomace	Antimicrobial [105], antioxidant [118,120], metabolic regulatory [105]		

**Table 6:** Main characteristics of products formulated in the included studies.

Food Matrix	By-Product	Main Bioactive Compounds	Functional Effects	Potential Administration Way	References
*Citrus sinensis* (L.) Osbeck	Pomace	Cyanide-3-glucoside Narirutin Hesperidin	Antioxidant Anti-inflammatory	Oral	[176]
*Olea europaea* L.	Mill wastewater	Hydroxytyrosol Tyrosol	Anti-inflammatory Antioxidant	Ophthalmic	[177]
*Psidium guajava*	Peel Pulp Seed	Apigenin Chlorogenic acid Myricetin	Antioxidant	Oral	[178]
*Cajanus cajan* (L.) Huth	Protein from mill waste	Protein hydrolysate	Antioxidant	Oral	[179]
*Prunus persica* (L.) Batsch *Solanum lycopersicum* L. *Olea europaea* L.	Thinning waste (nectarine) Peel (tomato) Leaves (olive)	Abscisic acid (nectarine) Carotenoids (tomato) Oleuropein (olive)	Antidiabetic	Oral	[180]
*Olea europaea* L.	Extruded olive paste (“Pâté”)	Hydroxytyrosol Tyrosol Verbascoside Luteolin	Antioxidant	Oral	[181]
*Prunus persica* (L.) Batsch	Thinning waste	Chlorogenic acid Neochlorogenic acid	Antioxidant Antidiabetic	Oral	[181]
*Olea europaea* L.	Mill wastewater	Hydroxytyrosol glycol Hydroxytyrosol Tyrosol Verbascoside Oleuropein	Antioxidant Improved skin hydration Reduced skin photo-irritation	Topical Oral	[182]
*Prunus dulcis* (Mill.) D. A, Webb	Peel	Catechin Procyanidin B3	Antioxidant Anti-inflammatory Antidiabetic	Oral	[183]
*Persea americana* Mill.	Seed	Catechin Hydroxybenzoic acid Rutin	Antioxidant	Oral	[184]
*Cynara cardunculus* L.	External bracts and stems	Caffeoylquinic acids Luteolin-7-glucoside	Antidiabetic	Oral	[185]
*Fragaria x ananassa* Duchesne *Rubus fruticosus* L. *Sambucus nigra* L. *Sorbus aucuparia* L.	Seeds (strawberry, blackberry, elderberry) Pomace (rowanberry)	β-sitosterol Proanthocyanidins Chlorophylls	Antioxidant UV radiation absorption Antimicrobial	Topical	[186]
*Corylus avellana* L.	Shell	Shikimic acid Ellagic acid Dihydroxy stearic acid Gallagic acid dilactone	Antioxidant Antidiabetic	Oral	[187]
*Plukenetia volubilis* L.	Seed shell	Phenolic compounds	Antimicrobial	Oral	[188]
*Olea europaea* L.	Leaves	Oleuropein	Antioxidant	Oral	[189]
*Spondias mombin* L.	Peel	Quercetin Ellagic acid Chlorogenic acid	Antioxidant	Oral	[190]

**Table 7:** Polymers extracted from agri-food by-products and their potential applications in food packaging.

By-Product	Polymer Matrix	Key Features	Reference
Citrus peel	Gelatin, glycerol, PLA/PHB, chitosan	Improved antioxidant and antimicrobial activity; enhanced UV barrier and mechanical properties	[246,247,248,249]
Spent coffee grounds	PLA, PHBV, pectin, κ-carrageenan	Improved thermal stability, antioxidant activity, enhanced mechanical strength	[250,251,252]
Grape pomace	Pectin, BioPBS, sodium alginate	Antioxidant and antimicrobial activity; UV screening; improved mechanical properties	[253,254,255]
Pomegranate peel	Chitosan, pectin, CMC	Antioxidant and antimicrobial activity; reduced water vapor permeability	[122,256,257]
Olive pomace/leaf	PHBV, PLA, CMC, chitosan	Antioxidant activity; mechanical reinforcement; antimicrobial activity	[258,259,260,261]

**Table 8:** Strategic roadmap for agri-food waste/by-product valorization.

Pillar	Key Barrier	Strategic Action	TRL Stage	TRL
Mechanistic Research	Limited understanding of in vivo effects	Identify bioactive compounds, mechanisms, and clinical studies	Basic/applied	2–4
Extraction and Formulation	Lack of scalable, standardized processes	Optimize green extraction and delivery systems	Pilot scale	4–6
Industrial Integration	High-cost variability of feedstock	Develop a modular biorefinery system to ensure raw material traceability	Scale-up	5–7
Regulatory Framework	Fragmented policies, absence of standards	Harmonize regulations, promote certifications	Regulatory	All TRLs
Cross-Sector Collaboration	Poor communication among disciplines	Launch educational campaign, enhance transparency, and sustainability storytelling	Deployment	8
Social Engagement	Law, consumer awareness, and potential skepticism		Deployment	8–9

## References

[1] Food and Agriculture Organization of the United Nations (FAO) Food Loss and Waste Database. https://www.fao.org/platform-food-loss-waste/flw-data/en/.

[2] Celeiro M., Lončarić A. (2024). Editorial: Plant Bioactive Compounds from Agro-Industrial by-Products for Improvement of Nutritional Quality of Foods. Front. Nutr..

[3] UN Environment Programme Emissions Gap Report 2024. https://www.unep.org/resources/emissions-gap-report-2024.

[4] Galanakis C.M. (2018). Sustainable Food Systems from Agriculture to Industry: Improving Production and Processing.

[5] Mirabella N., Castellani V., Sala S. (2014). Current Options for the Valorization of Food Manufacturing Waste: A Review. J. Clean. Prod..

[6] Shawky E., Gibbons S., Selim D.A. (2025). Bio-Sourcing from Byproducts: A Comprehensive Review of Bioactive Molecules in Agri-Food Waste (AFW) Streams for Valorization and Sustainable Applications. Bioresour. Technol..

[7] Spano M., Di Matteo G., Ingallina C., Ambroselli D., Carradori S., Gallorini M., Giusti A.M., Salvo A., Grosso M., Mannina L. (2022). Modulatory Properties of Food and Nutraceutical Components Targeting NLRP3 Inflammasome Activation. Nutrients.

[8] Graziani G., Gaspari A., Di Vaio C., Cirillo A., Ronca C.L., Grosso M., Ritieni A. (2021). Assessment of In Vitro Bioaccessibility of Polyphenols from Annurca, Limoncella, Red Delicious, and Golden Delicious Apples Using a Sequential Enzymatic Digestion Model. Antioxidants.

[9] Foti P., Romeo F.V., Caggia C. (2024). Olive Mill Wastewater as Booster to Produce a Blood Orange Juice with High Healthy Value. Food Saf. Health.

[10] Kurek M., Debeaufort F., Voilley A. (2024). Achievements in Applications of Antioxidants and Bioactive Compounds in Food: From Agriculture to Health Benefits. Antioxidants.

[11] Abbaspour N. (2024). Fermentation’s Pivotal Role in Shaping the Future of Plant-Based Foods: An Integrative Review of Fermentation Processes and Their Impact on Sensory and Health Benefits. Appl. Food Res..

[12] Boboua S.Y.B., Wen Q., Zhang L., Chen Y., Yu J., Chen P., Sun Y., Zheng T. (2024). Valorization of Animal Waste Proteins for Agricultural, Food Production, and Medicinal Applications. Front. Sustain. Food Syst..

[13] Tilman D., Clark M. (2014). Global Diets Link Environmental Sustainability and Human Health. Nature.

[14] Cappelli K., Ferlisi F., Mecocci S., Maranesi M., Trabalza-Marinucci M., Zerani M., Dal Bosco A., Acuti G. (2021). Dietary Supplementation of Olive Mill Waste Water Polyphenols in Rabbits: Evaluation of the Potential Effects on Hepatic Apoptosis, Inflammation and Metabolism through Rt-Qpcr Approach. Animals.

[15] Maqbool M.E., Farhan A., Qamar M.A. (2024). Global Impact of COVID-19 on Food Safety and Environmental Sustainability: Pathways to Face the Pandemic Crisis. Heliyon.

[16] Saini R.K., Khan M.I., Kumar V., Shang X., Lee J., Ko E. (2025). Bioactive Compounds of Agro-Industrial By-Products: Current Trends, Recovery, and Possible Utilization. Antioxidants.

[17] Cristofoli N.L., Lima A.R., Tchonkouang R.D.N., Quintino A.C., Vieira M.C. (2023). Advances in the Food Packaging Production from Agri-Food Waste and By-Products: Market Trends for a Sustainable Development. Sustainability.

[18] Lazaridis D.G., Andritsos N.D., Giannakas A.E., Karabagias I.K. (2025). Development and Valuation of Novel PLA-Based Biodegradable Packaging Materials Complemented with Food Waste of Plant and Animal Origin for Shelf-Life Extension of Selected Foods: Trends and Challenges. Sustainability.

[19] Salvo A., Masciulli F., Ambroselli D., Romano E., Ingallina C., Spano M., Di Matteo G., Giusti A.M., Di Sotto A., Percaccio E. (2024). Hydrolysates from Cauliflower and Artichoke Industrial Wastes as Biostimulants on Seed Germination and Seedling Growth: A Chemical and Biological Characterization. J. Sci. Food Agric..

[20] Chen C., Chaudhary A., Mathys A. (2020). Nutritional and Environmental Losses Embedded in Global Food Waste. Resour. Conserv. Recycl..

[21] Delgado L., Schuster M., Torero M. (2021). On the Origins of Food Loss. Appl. Econ. Perspect. Policy.

[22] Machado M., Silva S., Costa E.M. (2024). Byproducts as a Sustainable Source of Cosmetic Ingredients. Appl. Sci..

[23] Ahmad T., Esposito F., Cirillo T. (2024). Valorization of Agro-Food by-Products: Advancing Sustainability and Sustainable Development Goals 2030 through Functional Compounds Recovery. Food Biosci..

[24] Comunian T.A., Silva M.P., Souza C.J.F. (2021). The Use of Food By-Products as a Novel for Functional Foods: Their Use as Ingredients and for the Encapsulation Process. Trends Food Sci. Technol..

[25] Upadhyay S., Tiwari R., Kumar S., Gupta S.M., Kumar V., Rautela I., Kohli D., Rawat B.S., Kaushik R. (2023). Utilization of Food Waste for the Development of Composite Bread. Sustainability.

[26] Melini V., Melini F., Luziatelli F., Ruzzi M. (2020). Functional Ingredients from Agri-Food Waste: Effect of Inclusion Thereof on Phenolic Compound Content and Bioaccessibility in Bakery Products. Antioxidants.

[27] Klojdova I., Ngasakul N., Kozlu A., Baigts Allende D.K. (2024). Apple Pomace as a Functional Component of Sustainable Set-Type Yogurts. LWT.

[28] Calderón-Oliver M., López-Hernández L.H. (2022). Food Vegetable and Fruit Waste Used in Meat Products. Food Rev. Int..

[29] Torres-León C., Vicente A.A., Flores-López M.L., Rojas R., Serna-Cock L., Alvarez-Pérez O.B., Aguilar C.N. (2018). Edible Films and Coatings Based on Mango (Var. Ataulfo) by-Products to Improve Gas Transfer Rate of Peach. LWT.

[30] Ortiz L., Dorta E., Gloria Lobo M., Antonio González-Mendoza L., Díaz C., González M. (2017). Use of Banana Peel Extract to Stabilise Antioxidant Capacity and Sensory Properties of Orange Juice During Pasteurisation and Refrigerated Storage. Food Bioprocess Technol..

[31] Aguilar-Toalá J.E., Liceaga A.M. (2021). Cellular Antioxidant Effect of Bioactive Peptides and Molecular Mechanisms Underlying: Beyond Chemical Properties. Int. J. Food Sci. Technol..

[32] Goula A.M., Ververi M., Adamopoulou A., Kaderides K. (2017). Green Ultrasound-Assisted Extraction of Carotenoids from Pomegranate Wastes Using Vegetable Oils. Ultrason. Sonochem..

[33] Rodrigues D.B., Veríssimo L., Finimundy T., Rodrigues J., Oliveira I., Gonçalves J., Fernandes I.P., Barros L., Heleno S.A., Calhelha R.C. (2023). Chemical and Bioactive Screening of Green Polyphenol-Rich Extracts from Chestnut By-Products: An Approach to Guide the Sustainable Production of High-Added Value Ingredients. Foods.

[34] Sachs J.D., Lafortune G., Fuller G., Iablonovski G. (2025). Financing Sustainable Development to 2030 and Mid-Century. Sustainable Development Report 2025.

[35] Vicente-Zurdo D., Gómez-Mejía E., Morante-Zarcero S., Rosales-Conrado N., Sierra I. (2025). Analytical Strategies for Green Extraction, Characterization, and Bioactive Evaluation of Polyphenols, Tocopherols, Carotenoids, and Fatty Acids in Agri-Food Bio-Residues. Molecules.

[36] Sonawane A., Pathak S., Pradhan R.C. (2021). Bioactive Compounds in Bael Fruit Pulp Waste: Ultrasound-Assisted Extraction, Characterization, Modeling, and Optimization Approaches. Biointerface Res. Appl. Chem..

[37] Wang W., Ma X., Xu Y., Cao Y., Jiang Z., Ding T., Ye X., Liu D. (2015). Ultrasound-Assisted Heating Extraction of Pectin from Grapefruit Peel: Optimization and Comparison with the Conventional Method. Food Chem..

[38] Khalfi A., Garrigós M.C., Ramos M., Jiménez A. (2024). Optimization of the Microwave-Assisted Extraction Conditions for Phenolic Compounds from Date Seeds. Foods.

[39] Solaberrieta I., Mellinas C., Jiménez A., Garrigós M.C. (2022). Recovery of Antioxidants from Tomato Seed Industrial Wastes by Microwave-Assisted and Ultrasound-Assisted Extraction. Foods.

[40] Varadharajan V., Shanmugam S., Ramaswamy A. (2017). Model Generation and Process Optimization of Microwave-Assisted Aqueous Extraction of Anthocyanins from Grape Juice Waste. J. Food Process Eng..

[41] Alchera F., Ginepro M., Giacalone G. (2024). Microwave-Assisted Extraction (MAE) of Bioactive Compounds from Blueberry By-Products Using a Sugar-Based NADES: A Novelty in Green Chemistry. LWT.

[42] Tomasi I.T., Santos S.C.R., Boaventura R.A.R., Botelho C.M.S. (2023). Optimization of Microwave-Assisted Extraction of Phenolic Compounds from Chestnut Processing Waste Using Response Surface Methodology. J. Clean. Prod..

[43] Cardullo N., Leanza M., Muccilli V., Tringali C. (2021). Valorization of Agri-Food Waste from Pistachio Hard Shells: Extraction of Polyphenols as Natural Antioxidants. Resources.

[44] Nieto J.A., Santoyo S., Prodanov M., Reglero G., Jaime L. (2020). Valorisation of Grape Stems as a Source of Phenolic Antioxidants by Using a Sustainable Extraction Methodology. Foods.

[45] Pereira D.T.V., Tarone A.G., Cazarin C.B.B., Barbero G.F., Martínez J. (2019). Pressurized Liquid Extraction of Bioactive Compounds from Grape Marc. J. Food Eng..

[46] Ruiz-Aceituno L., García-Sarrió M.J., Alonso-Rodriguez B., Ramos L., Sanz M.L. (2016). Extraction of Bioactive Carbohydrates from Artichoke (*Cynara scolymus* L.) External Bracts Using Microwave Assisted Extraction and Pressurized Liquid Extraction. Food Chem..

[47] Solana M., Boschiero I., Dall’Acqua S., Bertucco A. (2015). A Comparison between Supercritical Fluid and Pressurized Liquid Extraction Methods for Obtaining Phenolic Compounds from *Asparagus officinalis* L.. J. Supercrit. Fluids.

[48] Çam M., Hişil Y. (2010). Pressurised Water Extraction of Polyphenols from Pomegranate Peels. Food Chem..

[49] de Andrade Maia F., Fasolin L.H. (2025). Recovery of Bioactive Compounds from Pineapple Waste through High-Pressure Technologies. J. Supercrit. Fluids.

[50] Figueroa J.G., Borrás-Linares I., Lozano-Sánchez J., Quirantes-Piné R., Segura-Carretero A. (2018). Optimization of Drying Process and Pressurized Liquid Extraction for Recovery of Bioactive Compounds from Avocado Peel By-Product. Electrophoresis.

[51] Katsinas N., Bento Da Silva A., Enríquez-De-Salamanca A., Fernández N., Bronze M.R., Rodríguez-Rojo S. (2021). Pressurized Liquid Extraction Optimization from Supercritical Defatted Olive Pomace: A Green and Selective Phenolic Extraction Process. ACS Sustain. Chem. Eng..

[52] Lachos-Perez D., Baseggio A.M., Mayanga-Torres P.C., Maróstica M.R., Rostagno M.A., Martínez J., Forster-Carneiro T. (2018). Subcritical Water Extraction of Flavanones from Defatted Orange Peel. J. Supercrit. Fluids.

[53] Loarce L., Oliver-Simancas R., Marchante L., Díaz-Maroto M.C., Alañón M.E. (2021). Modifiers Based on Natural Deep Eutectic Mixtures to Enhance Anthocyanins Isolation from Grape Pomace by Pressurized Hot Water Extraction. LWT.

[54] Liew S.Q., Ngoh G.C., Yusoff R., Teoh W.H. (2018). Acid and Deep Eutectic Solvent (DES) Extraction of Pectin from Pomelo (*Citrus grandis* (L.) Osbeck) Peels. Biocatal. Agric. Biotechnol..

[55] Loarce L., Oliver-Simancas R., Marchante L., Díaz-Maroto M.C., Alañón M.E. (2020). Implementation of Subcritical Water Extraction with Natural Deep Eutectic Solvents for Sustainable Extraction of Phenolic Compounds from Winemaking By-Products. Food Res. Int..

[56] Grassino A.N., Pedisić S., Dragović-Uzelac V., Karlović S., Ježek D., Bosiljkov T. (2020). Insight into High-Hydrostatic Pressure Extraction of Polyphenols from Tomato Peel Waste. Plant Foods Hum. Nutr..

[57] Okur I., Namlı S., Oztop M.H., Alpas H. (2023). High-Pressure-Assisted Extraction of Phenolic Compounds from Olive Leaves: Optimization and Comparison with Conventional Extraction. ACS Food Sci. Technol..

[58] Xie F., Zhang W., Lan X., Gong S., Wu J., Wang Z. (2018). Effects of High Hydrostatic Pressure and High Pressure Homogenization Processing on Characteristics of Potato Peel Waste Pectin. Carbohydr. Polym..

[59] Scaglia B., D’Incecco P., Squillace P., Dell’Orto M., De Nisi P., Pellegrino L., Botto A., Cavicchi C., Adani F. (2020). Development of a Tomato Pomace Biorefinery Based on a CO_2_-Supercritical Extraction Process for the Production of a High Value Lycopene Product, Bioenergy and Digestate. J. Clean. Prod..

[60] Solaberrieta I., Mellinas A.C., Espagnol J., Hamzaoui M., Jiménez A., Garrigós M.C. (2022). Valorization of Tomato Seed By-Products as a Source of Fatty Acids and Bioactive Compounds by Using Advanced Extraction Techniques. Foods.

[61] Trabelsi D., Aydi A., Zibetti A.W., Della Porta G., Scognamiglio M., Cricchio V., Langa E., Abderrabba M., Mainar A.M. (2016). Supercritical Extraction from Citrus Aurantium Amara Peels Using CO_2_ with Ethanol as Co-Solvent. J. Supercrit. Fluids.

[62] Jerkovic I., Druzic J., Marijanovic Z., Gugic M., Jokić S.D., Roje M. (2015). GC-FID/MS Profiling of Supercritical CO_2_ extracts of Peels from *Citrus aurantium*, *C. sinensis* Cv. Washington Navel, *C. sinensis* Cv. Tarocco and *C. sinensis* Cv. Doppio Sanguigno from Dubrovnik Area (Croatia). Nat. Prod. Commun..

[63] Mora J.J., Tavares H.M., Curbelo R., Dellacassa E., Cassel E., Apel M.A., von Poser G.L., Vargas R.M.F. (2025). Supercritical Fluid Extraction of Coumarins and Flavonoids from Citrus Peel. J. Supercrit. Fluids.

[64] Romano R., De Luca L., Aiello A., Rossi D., Pizzolongo F., Masi P. (2022). Bioactive Compounds Extracted by Liquid and Supercritical Carbon Dioxide from Citrus Peels. Int. J. Food Sci. Technol..

[65] Ortiz-Sanchez M., Agudelo-Patiño T., Cardona Alzate C.A. (2024). Maximizing the Hesperidin Extraction Using Supercritical Carbon Dioxide and Ethanol: Theoretical Prediction and Experimental Results. Processes.

[66] Luengo E., Álvarez I., Raso J. (2014). Improving Carotenoid Extraction from Tomato Waste by Pulsed Electric Fields. Front. Nutr..

[67] Andreou V., Dimopoulos G., Dermesonlouoglou E., Taoukis P. (2020). Application of Pulsed Electric Fields to Improve Product Yield and Waste Valorization in Industrial Tomato Processing. J. Food Eng..

[68] Brianceau S., Turk M., Vitrac X., Vorobiev E. (2015). Combined Densification and Pulsed Electric Field Treatment for Selective Polyphenols Recovery from Fermented Grape Pomace. Innov. Food Sci. Emerg. Technol..

[69] Carpentieri S., Ferrari G., Pataro G. (2023). Pulsed Electric Fields-Assisted Extraction of Valuable Compounds from Red Grape Pomace: Process Optimization Using Response Surface Methodology. Front. Nutr..

[70] Deng Y., Ju T., Xi J. (2018). Circulating Polyphenols Extraction System with High-Voltage Electrical Discharge: Design and Performance Evaluation. ACS Sustain. Chem. Eng..

[71] Xi J., He L., Yan L.-g. (2017). Continuous Extraction of Phenolic Compounds from Pomegranate Peel Using High Voltage Electrical Discharge. Food Chem..

[72] Shen L., Pang S., Zhong M., Sun Y., Qayum A., Liu Y., Rashid A., Xu B., Liang Q., Ma H. (2023). A Comprehensive Review of Ultrasonic Assisted Extraction (UAE) for Bioactive Components: Principles, Advantages, Equipment, and Combined Technologies. Ultrason. Sonochem..

[73] Mellinas C., Solaberrieta I., Pelegrín C.J., Jiménez A., Garrigós M.C. (2022). Valorization of Agro-Industrial Wastes by Ultrasound-Assisted Extraction as a Source of Proteins, Antioxidants and Cutin: A Cascade Approach. Antioxidants.

[74] Kewlani P., Singh L., Singh B., Bhatt I.D. (2022). Sustainable Extraction of Phenolics and Antioxidant Activities from Prinsepia Utilis Byproducts for Alleviating Aging and Oxidative Stress. Sustain. Chem. Pharm..

[75] Mármol I., Quero J., Ibarz R., Ferreira-Santos P., Teixeira J.A., Rocha C.M.R., Pérez-Fernández M., García-Juiz S., Osada J., Martín-Belloso O. (2021). Valorization of Agro-Food by-Products and Their Potential Therapeutic Applications. Food Bioprod. Process..

[76] Boateng I.D., Clark K. (2024). Trends in Extracting Agro-Byproducts’ Phenolics Using Non-Thermal Technologies and Their Combinative Effect: Mechanisms, Potentials, Drawbacks, and Safety Evaluation. Food Chem..

[77] Gil-Martín E., Forbes-Hernández T., Romero A., Cianciosi D., Giampieri F., Battino M. (2022). Influence of the Extraction Method on the Recovery of Bioactive Phenolic Compounds from Food Industry By-Products. Food Chem..

[78] Ingallina C., Maccelli A., Spano M., Di Matteo G., Di Sotto A., Giusti A.M., Vinci G., Di Giacomo S., Rapa M., Ciano S. (2020). Chemico-Biological Characterization of Torpedino Di Fondi® Tomato Fruits: A Comparison with San Marzano Cultivar at Two Ripeness Stages. Antioxidants.

[79] Spano M., Di Matteo G., Fernandez Retamozo C.A., Lasalvia A., Ruggeri M., Sandri G., Cordeiro C., Sousa Silva M., Totaro Fila C., Garzoli S. (2023). A Multimethodological Approach for the Chemical Characterization of Edible Insects: The Case Study of *Acheta domesticus*. Foods.

[80] Socas-Rodríguez B., Álvarez-Rivera G., Valdés A., Ibáñez E., Cifuentes A. (2021). Food By-Products and Food Wastes: Are They Safe Enough for Their Valorization?. Trends Food Sci. Technol..

[81] López-Salazar H., Camacho-Díaz B.H., Arenas Ocampo M.L., Jiménez-Aparicio A.R. (2023). Microwave-Assisted Extraction of Functional Compounds from Plants: A Review. BioResources.

[82] Sparr Eskilsson C., Björklund E. (2000). Analytical-Scale Microwave-Assisted Extraction. J. Chromatogr. A.

[83] Uddin M.S., Ferdosh S., Haque Akanda M.J., Ghafoor K., Rukshana A.H., Ali M.E., Kamaruzzaman B.Y., Fauzi M.B., Hadijah S., Shaarani S. (2018). Techniques for the Extraction of Phytosterols and Their Benefits in Human Health: A Review. Sep. Sci. Technol..

[84] Kaufmann B., Christen P. (2002). Recent Extraction Techniques for Natural Products: Microwave-Assisted Extraction and Pressurised Solvent Extraction. Phytochem. Anal..

[85] Haffizi M., Sulaiman S., Noraini Jimat D., Amid A. (2020). Review: A Comparison of Conditions for the Extraction of Vegetable and Essential Oils Via Microwave-Assisted Extraction. IOP Conf. Ser. Mater. Sci. Eng..

[86] Perino S., Petitcolas E., de la Guardia M., Chemat F. (2013). Portable Microwave Assisted Extraction: An Original Concept for Green Analytical Chemistry. J. Chromatogr. A.

[87] Shu Y.Y., Lai T.L., Lin H.S., Yang T.C., Chang C.P. (2003). Study of Factors Affecting on the Extraction Efficiency of Polycyclic Aromatic Hydrocarbons from Soils Using Open-Vessel Focused Microwave-Assisted Extraction. Chemosphere.

[88] Ferrara D., Beccaria M., Cordero C.E., Purcaro G. (2023). Microwave-Assisted Extraction in Closed Vessel in Food Analysis. J. Sep. Sci..

[89] Rhazi N., Hannache H., Oumam M., Sesbou A., Charrier B., Pizzi A., Charrier-El Bouhtoury F. (2019). Green Extraction Process of Tannins Obtained from Moroccan Acacia Mollissima Barks by Microwave: Modeling and Optimization of the Process Using the Response Surface Methodology RSM. Arab. J. Chem..

[90] Taghian Dinani S., van der Goot A.J. (2023). Challenges and Solutions of Extracting Value-Added Ingredients from Fruit and Vegetable by-Products: A Review. Crit. Rev. Food Sci. Nutr..

[91] Jiménez-Moreno N., Esparza I., Bimbela F., Gandía L.M., Ancín-Azpilicueta C. (2020). Valorization of Selected Fruit and Vegetable Wastes as Bioactive Compounds: Opportunities and Challenges. Crit. Rev. Environ. Sci. Technol..

[92] Mendiola J.A., Herrero M., Cifuentes A., Ibañez E. (2007). Use of Compressed Fluids for Sample Preparation: Food Applications. J. Chromatogr. A.

[93] Arshadi M., Attard T.M., Lukasik R.M., Brncic M., Da Costa Lopes A.M., Finell M., Geladi P., Gerschenson L.N., Gogus F., Herrero M. (2016). Pre-Treatment and Extraction Techniques for Recovery of Added Value Compounds from Wastes throughout the Agri-Food Chain. Green Chem..

[94] Liew S.Q., Teoh W.H., Tan C.K., Yusoff R., Ngoh G.C. (2018). Subcritical Water Extraction of Low Methoxyl Pectin from Pomelo (*Citrus grandis* (L.) Osbeck) Peels. Int. J. Biol. Macromol..

[95] Carpentieri S., Soltanipour F., Ferrari G., Pataro G., Donsì F. (2021). Emerging Green Techniques for the Extraction of Antioxidants from Agri-Food By-Products as Promising Ingredients for the Food Industry. Antioxidants.

[96] Dai Y., van Spronsen J., Witkamp G.J., Verpoorte R., Choi Y.H. (2013). Natural Deep Eutectic Solvents as New Potential Media for Green Technology. Anal. Chim. Acta.

[97] Dheyab A.S., Bakar M.F.A., Alomar M., Sabran S.F., Hanafi A.F.M., Mohamad A. (2021). Deep Eutectic Solvents (DESs) as Green Extraction Media of Beneficial Bioactive Phytochemicals. Separations.

[98] Balaraman H., Selvasembian R., Rangarajan V., Rathnasamy S. (2021). Sustainable and Green Engineering Insights on Deep Eutectic Solvents toward the Extraction of Nutraceuticals. ACS Sustain. Chem. Eng..

[99] Chemat F., Abert Vian M., Fabiano-Tixier A.S., Nutrizio M., Režek Jambrak A., Munekata P.E.S., Lorenzo J.M., Barba F.J., Binello A., Cravotto G. (2020). A Review of Sustainable and Intensified Techniques for Extraction of Food and Natural Products. Green Chem..

[100] Naliyadhara N., Kumar A., Girisa S., Daimary U.D., Hegde M., Kunnumakkara A.B. (2022). Pulsed Electric Field (PEF): Avant-Garde Extraction Escalation Technology in Food Industry. Trends Food Sci. Technol..

[101] Li Z., Fan Y., Xi J. (2019). Recent Advances in High Voltage Electric Discharge Extraction of Bioactive Ingredients from Plant Materials. Food Chem..

[102] Moro K.I.B., Bender A.B.B., da Silva L.P., Penna N.G. (2021). Green Extraction Methods and Microencapsulation Technologies of Phenolic Compounds from Grape Pomace: A Review. Food Bioprocess Technol..

[103] McNutt J., He Q. (2019). (Sophia) Spent Coffee Grounds: A Review on Current Utilization. J. Ind. Eng. Chem..

[104] Shaban N.Z., El-Kersh M.A.L., El-Rashidy F.H., Habashy N.H. (2013). Protective Role of *Punica granatum* (Pomegranate) Peel and Seed Oil Extracts on Diethylnitrosamine and Phenobarbital-Induced Hepatic Injury in Male Rats. Food Chem..

[105] Sha S.P., Modak D., Sarkar S., Roy S.K., Sah S.P., Ghatani K., Bhattacharjee S. (2023). Fruit Waste: A Current Perspective for the Sustainable Production of Pharmacological, Nutraceutical, and Bioactive Resources. Front. Microbiol..

[106] Zhang P., Liu H., Yu Y., Peng S., Zhu S. (2024). Role of Curcuma Longae Rhizoma in Medical Applications: Research Challenges and Opportunities. Front. Pharmacol..

[107] Liczbiński P., Michałowicz J., Bukowska B. (2020). Molecular Mechanism of Curcumin Action in Signaling Pathways: Review of the Latest Research. Phytother. Res..

[108] Di Sotto A., Di Giacomo S., Amatore D., Locatelli M., Vitalone A., Toniolo C., Rotino G.L., Lo Scalzo R., Palamara A.T., Marcocci M.E. (2018). A Polyphenol Rich Extract from *Solanum melongena* L. DR2 Peel Exhibits Antioxidant Properties and Anti-Herpes Simplex Virus Type 1 Activity In Vitro. Molecules.

[109] Dong H., Sargent L.J., Chatzidiakou Y., Saunders C., Harkness L., Bordenave N., Rowland I., Spencer J.P.E., Lovegrove J.A. (2016). Orange Pomace Fibre Increases a Composite Scoring of Subjective Ratings of Hunger and Fullness in Healthy Adults. Appetite.

[110] Choleva M., Matalliotaki E., Antoniou S., Asimomyti E., Drouka A., Stefani M., Yannakoulia M., Fragopoulou E. (2023). Postprandial Metabolic and Oxidative Stress Responses to Grape Pomace Extract in Healthy Normal and Overweight/Obese Women: A Randomized, Double-Blind, Placebo-Controlled Crossover Study. Nutrients.

[111] López-Yerena A., Domínguez-López I., Abuhabib M.M., Lamuela-Raventós R.M., Vallverdú-Queralt A., Pérez M. (2023). Tomato Wastes and By-Products: Upcoming Sources of Polyphenols and Carotenoids for Food, Nutraceutical, and Pharma Applications. Crit. Rev. Food Sci. Nutr..

[112] Van Hung P. (2016). Phenolic Compounds of Cereals and Their Antioxidant Capacity. Crit. Rev. Food Sci. Nutr..

[113] Weng Y.X., Wang H.C., Chu Y.L., Wu Y.Z., Liao J.A., Su Z.Y. (2024). Essential Oil from Citrus Depressa Peel Exhibits Antimicrobial, Antioxidant and Cancer Chemopreventive Effects. J. Sci. Food Agric..

[114] Xiang Q., Li M., Wen J., Ren F., Yang Z., Jiang X., Chen Y. (2022). The Bioactivity and Applications of Pomegranate Peel Extract: A Review. J. Food Biochem..

[115] Poprac P., Jomova K., Simunkova M., Kollar V., Rhodes C.J., Valko M. (2017). Targeting Free Radicals in Oxidative Stress-Related Human Diseases. Trends Pharmacol. Sci..

[116] Ruíz-Delgado A., Roccamante M.A., Oller I., Agüera A., Malato S. (2019). Natural Chelating Agents from Olive Mill Wastewater to Enable Photo-Fenton-like Reactions at Natural PH. Catal. Today.

[117] Khanam A., Ahmad S., Husain A., Rehman S., Farooqui A., Yusuf M.A. (2020). Glycation and Antioxidants: Hand in the Glove of Antiglycation and Natural Antioxidants. Curr. Protein Pept. Sci..

[118] Anwar S., Khan S., Almatroudi A., Khan A.A., Alsahli M.A., Almatroodi S.A., Rahmani A.H. (2021). A Review on Mechanism of Inhibition of Advanced Glycation End Products Formation by Plant Derived Polyphenolic Compounds. Mol. Biol. Rep..

[119] Coelho O.G.L., Ribeiro P.V.M., Alfenas R.d.C.G. (2023). Can Grape Polyphenols Affect Glycation Markers? A Systematic Review. Crit. Rev. Food Sci. Nutr..

[120] Sharma S., Rana S., Patial V., Gupta M., Bhushan S., Padwad Y.S. (2016). Antioxidant and Hepatoprotective Effect of Polyphenols from Apple Pomace Extract via Apoptosis Inhibition and Nrf2 Activation in Mice. Hum. Exp. Toxicol..

[121] Alves I., Araújo E.M.Q., Dalgaard L.T., Singh S., Børsheim E., Carvalho E. (2025). Protective Effects of Sulforaphane Preventing Inflammation and Oxidative Stress to Enhance Metabolic Health: A Narrative Review. Nutrients.

[122] Kumar N., Daniloski D., Pratibha, Neeraj, D’Cunha N.M., Naumovski N., Petkoska A.T. (2022). Pomegranate Peel Extract—A Natural Bioactive Addition to Novel Active Edible Packaging. Food Res. Int..

[123] Behl T., Rana T., Alotaibi G.H., Shamsuzzaman M., Naqvi M., Sehgal A., Singh S., Sharma N., Almoshari Y., Abdellatif A.A.H. (2022). Polyphenols Inhibiting MAPK Signalling Pathway Mediated Oxidative Stress and Inflammation in Depression. Biomed. Pharmacother..

[124] Treasure K., Harris J., Williamson G. (2023). Exploring the Anti-Inflammatory Activity of Sulforaphane. Immunol. Cell Biol..

[125] Zhang T., Ma C., Zhang Z., Zhang H., Hu H. (2021). NF-ΚB Signaling in Inflammation and Cancer. MedComm.

[126] He Y., Sun Z., Bai J.Y., Zhang Y., Qian Y., Zhao X., Chen S. (2023). Citrus Peel Polyphenols Alleviate Intestinal Inflammation in Mice with Dextran Sulfate Sodium-Induced Acute Colitis. Heliyon.

[127] Fadel A., Mahmoud A.M., Ashworth J.J., Li W., Ng Y.L., Plunkett A. (2018). Health-Related Effects and Improving Extractability of Cereal Arabinoxylans. Int. J. Biol. Macromol..

[128] Franco-Robles E., López M.G. (2015). Implication of Fructans in Health: Immunomodulatory and Antioxidant Mechanisms. Sci. World J..

[129] Ney L.M., Wipplinger M., Grossmann M., Engert N., Wegner V.D., Mosig A.S. (2023). Short Chain Fatty Acids: Key Regulators of the Local and Systemic Immune Response in Inflammatory Diseases and Infections. Open Biol..

[130] Albini A., Albini F., Corradino P., Dugo L., Calabrone L., Noonan D.M. (2023). From Antiquity to Contemporary Times: How Olive Oil By-Products and Waste Water Can Contribute to Health. Front. Nutr..

[131] Azmat F., Safdar M., Ahmad H., Khan M.R.J., Abid J., Naseer M.S., Aggarwal S., Imran A., Khalid U., Zahra S.M. (2024). Phytochemical Profile, Nutritional Composition of Pomegranate Peel and Peel Extract as a Potential Source of Nutraceutical: A Comprehensive Review. Food Sci. Nutr..

[132] Coelho M.C., Rodrigues A.S., Teixeira J.A., Pintado M.E. (2023). Integral Valorisation of Tomato By-Products towards Bioactive Compounds Recovery: Human Health Benefits. Food Chem..

[133] Albini A., Festa M.M.G., Ring N., Baci D., Rehman M., Finzi G., Sessa F., Zacchigna S., Bruno A., Noonan D.M. (2021). A Polyphenol-Rich Extract of Olive Mill Wastewater Enhances Cancer Chemotherapy Effects, While Mitigating Cardiac Toxicity. Front. Pharmacol..

[134] Habib H.M., El-Fakharany E.M., Kheadr E., Ibrahim W.H. (2022). Grape Seed Proanthocyanidin Extract Inhibits DNA and Protein Damage and Labile Iron, Enzyme, and Cancer Cell Activities. Sci. Rep..

[135] Di Sotto A., Vecchiato M., Abete L., Toniolo C., Giusti A.M., Mannina L., Locatelli M., Nicoletti M., Di Giacomo S. (2018). *Capsicum annuum* L. Var. Cornetto Di Pontecorvo PDO: Polyphenolic Profile and In Vitro Biological Activities. J. Funct. Foods.

[136] Durmus N., Kilic-Akyilmaz M. (2023). Bioactivity of Non-Extractable Phenolics from Lemon Peel Obtained by Enzyme and Ultrasound Assisted Extractions. Food Biosci..

[137] Bai R., Yuan C., Wang T., Liu L., Li J., Lai Y., Li H., Chen Z., Li C., Ke D. (2020). Apple Pomace and Rosemary Extract Ameliorates Hepatic Steatosis in Fructose-Fed Rats: Association with Enhancing Fatty Acid Oxidation and Suppressing Inflammation. Exp. Ther. Med..

[138] Naveed M., Hejazi V., Abbas M., Kamboh A.A., Khan G.J., Shumzaid M., Ahmad F., Babazadeh D., FangFang X., Modarresi-Ghazani F. (2018). Chlorogenic Acid (CGA): A Pharmacological Review and Call for Further Research. Biomed. Pharmacother..

[139] Kumari K., Nagar S., Goyal S., Maan S., Chugh V., Kumar V., Kharor N. (2024). Xylooligosaccharide Production from Lignocellulosic Biomass and Their Health Benefits as Prebiotics. Biochem. Res. Int..

[140] Zurbau A., Noronha J.C., Khan T.A., Sievenpiper J.L., Wolever T.M.S. (2021). The Effect of Oat β-Glucan on Postprandial Blood Glucose and Insulin Responses: A Systematic Review and Meta-Analysis. Eur. J. Clin. Nutr..

[141] Blasi F., Ianni F., Mangiapelo L., Pinna N., Cossignani L. (2023). In Vitro Anti-Obesity Activity by Pancreatic Lipase Inhibition—Simple HPLC Approach Using EVOO as Natural Substrate. J. Sci. Food Agric..

[142] Liu T.T., Liu X.T., Chen Q.X., Shi Y. (2020). Lipase Inhibitors for Obesity: A Review. Biomed. Pharmacother..

[143] Binou P., Yanni A.E., Stergiou A., Karavasilis K., Konstantopoulos P., Perrea D., Tentolouris N., Karathanos V.T. (2021). Enrichment of Bread with Beta-Glucans or Resistant Starch Induces Similar Glucose, Insulin and Appetite Hormone Responses in Healthy Adults. Eur. J. Nutr..

[144] Warrilow A., Mellor D., McKune A., Pumpa K. (2019). Dietary Fat, Fibre, Satiation, and Satiety—A Systematic Review of Acute Studies. Eur. J. Clin. Nutr..

[145] Mah E., Liska D.A.J., Goltz S., Chu Y.F. (2023). The Effect of Extracted and Isolated Fibers on Appetite and Energy Intake: A Comprehensive Review of Human Intervention Studies. Appetite.

[146] Mozaffarian D., Aro A., Willett W.C. (2009). Health Effects of Trans-Fatty Acids: Experimental and Observational Evidence. Eur. J. Clin. Nutr..

[147] Lau K.Q., Sabran M.R., Shafie S.R. (2021). Utilization of Vegetable and Fruit By-Products as Functional Ingredient and Food. Front. Nutr..

[148] Aliakbarian B., Casale M., Paini M., Casazza A.A., Lanteri S., Perego P. (2015). Production of a Novel Fermented Milk Fortified with Natural Antioxidants and Its Analysis by NIR Spectroscopy. LWT.

[149] Haque A., Ahmad S., Azad Z.R.A.A., Adnan M., Ashraf S.A. (2023). Incorporating Dietary Fiber from Fruit and Vegetable Waste in Meat Products: A Systematic Approach for Sustainable Meat Processing and Improving the Functional, Nutritional and Health Attributes. PeerJ.

[150] Iriondo-Dehond M., Miguel E., Del Castillo M.D. (2018). Food Byproducts as Sustainable Ingredients for Innovative and Healthy Dairy Foods. Nutrients.

[151] Lucera A., Costa C., Marinelli V., Saccotelli M.A., Del Nobile M.A., Conte A. (2018). Fruit and Vegetable By-Products to Fortify Spreadable Cheese. Antioxidants.

[152] Issar K., Sharma P.C., Gupta A. (2017). Utilization of Apple Pomace in the Preparation of Fiber-Enriched Acidophilus Yoghurt. J. Food Process. Preserv..

[153] Shinali T.S., Zhang Y., Altaf M., Nsabiyeze A., Han Z., Shi S., Shang N. (2024). The Valorization of Wastes and Byproducts from Cruciferous Vegetables: A Review on the Potential Utilization of Cabbage, Cauliflower, and Broccoli Byproducts. Foods.

[154] Coman V., Teleky B.E., Mitrea L., Martău G.A., Szabo K., Călinoiu L.F., Vodnar D.C. (2020). Bioactive Potential of Fruit and Vegetable Wastes. Adv. Food Nutr. Res..

[155] Rațu R.N., Veleșcu I.D., Stoica F., Usturoi A., Arsenoaia V.N., Crivei I.C., Postolache A.N., Lipșa F.D., Filipov F., Florea A.M. (2023). Application of Agri-Food By-Products in the Food Industry. Agriculture.

[156] Betrouche A., Estivi L., Colombo D., Pasini G., Benatallah L., Brandolini A., Hidalgo A. (2022). Antioxidant Properties of Gluten-Free Pasta Enriched with Vegetable By-Products. Molecules.

[157] Tlais A.Z.A., Maria Fiorino G., Polo A., Filannino P., Di Cagno R. (2020). High-Value Compounds in Fruit, Vegetable and Cereal By-products: An Overview of Potential Sustainable Reuse and Exploitation. Molecules.

[158] Temple N.J. (2022). A Rational Definition for Functional Foods: A Perspective. Front. Nutr..

[159] Piercy E., Verstraete W., Ellis P.R., Banks M., Rockström J., Smith P., Witard O.C., Hallett J., Hogstrand C., Knott G. (2022). A Sustainable Waste-to-Protein System to Maximise Waste Resource Utilisation for Developing Food- and Feed-Grade Protein Solutions. Green Chem..

[160] Hussain M.A., Bekhit A.E.D.A. (2023). Innovative Foods: The Future Food Supply, Nutrition and Health. Foods.

[161] Ullagaddi R. (2025). Food Waste Upcycling and Functional Foods: Innovations for Health and Sustainability. Afr. J. Biomed. Res..

[162] Di Nunzio M., Picone G., Pasini F., Chiarello E., Caboni M.F., Capozzi F., Gianotti A., Bordoni A. (2020). Olive Oil By-Product as Functional Ingredient in Bakery Products. Influence of Processing and Evaluation of Biological Effects. Food Res. Int..

[163] Breschi C., D’Agostino S., Meneguzzo F., Zabini F., Chini J., Lovatti L., Tagliavento L., Guerrini L., Bellumori M., Cecchi L. (2024). Can a Fraction of Flour and Sugar Be Replaced with Fruit By-Product Extracts in a Gluten-Free and Vegan Cookie Recipe?. Molecules.

[164] D’Ambra K., Trovato R., Minelli G., Cattivelli A., Zannini M., Tagliazucchi D., Tabasso S., Lo Fiego D. (2025). Pietro Hazelnut Skin Polyphenolic Green Extract as a Promising Natural Antioxidant in Pork Burgers: Assessment of Quality Parameters and Consumer Acceptance. Food Res. Int..

[165] Gottardi D., Siroli L., Braschi G., D’Alessandro M., Vannini L., Patrignani F., Lanciotti R. (2025). Surface Application and Impact of Yarrowia Lipolytica Grown in Cheese Whey as Adjunct Culture for Innovative and Fast-Ripening Caciotta-like Cheeses. Int. J. Food Microbiol..

[166] González-Montelongo R., Gloria Lobo M., González M. (2010). Antioxidant Activity in Banana Peel Extracts: Testing Extraction Conditions and Related Bioactive Compounds. Food Chem..

[167] Ibrahim N.I., Shahar F.S., Hameed Sultan M.T., Md Shah A.U., Azrie Safri S.N., Mat Yazik M.H. (2021). Overview of Bioplastic Introduction and Its Applications in Product Packaging. Coatings.

[168] Bajerska J., Mildner-Szkudlarz S., Górnaś P., Seglina D. (2016). The Effects of Muffins Enriched with Sour Cherry Pomace on Acceptability, Glycemic Response, Satiety and Energy Intake: A Randomized Crossover Trial. J. Sci. Food Agric..

[169] Younis K., Ahmad S., Malik M.A. (2021). Mosambi Peel Powder Incorporation in Meat Products: Effect on Physicochemical Properties and Shelf Life Stability. Appl. Food Res..

[170] Nam J.K., Lee J.Y., Jang H.W. (2024). Quality Characteristics and Volatile Compounds of Plant-Based Patties Supplemented with Biji Powder. Food Chem. X.

[171] Olufunso A.E., Cyril N.C., Grace T.O., Olajide A.A., Kehinde O.O., Martha O.D., Gibson C.O., Olusola A.O. (2018). Sensory Evaluation of Meat of Broiler Poultry Birds Fed with Tomato-Supplemented Feed. Technol. Sci. Am. Sci. Res. J. Eng..

[172] Rafiq S., Singh B., Gat Y. (2019). Effect of Different Drying Techniques on Chemical Composition, Color and Antioxidant Properties of Kinnow (*Citrus reticulata*) Peel. J. Food Sci. Technol..

[173] Amofa-Diatuo T., Anang D.M., Barba F.J., Tiwari B.K. (2017). Development of New Apple Beverages Rich in Isothiocyanates by Using Extracts Obtained from Ultrasound-Treated Cauliflower by-Products: Evaluation of Physical Properties and Consumer Acceptance. J. Food Compos. Anal..

[174] Wedamulla N.E., Fan M., Choi Y.J., Kim E.K. (2022). Citrus Peel as a Renewable Bioresource: Transforming Waste to Food Additives. J. Funct. Foods.

[175] Morales D., Gutiérrez-Pensado R., Bravo F.I., Muguerza B. (2023). Novel Kombucha Beverages with Antioxidant Activity Based on Fruits as Alternative Substrates. LWT.

[176] Lauro M.R., Crasci L., Carbone C., Aquino R.P., Panico A.M., Puglisi G. (2015). Encapsulation of a Citrus By-Product Extract: Development, Characterization and Stability Studies of a Nutraceutical with Antioxidant and Metalloproteinases Inhibitory Activity. LWT.

[177] Di Mauro M.D., Fava G., Spampinato M., Aleo D., Melilli B., Saita M.G., Centonze G., Maggiore R., D’antona N. (2019). Polyphenolic Fraction from Olive Mill Wastewater: Scale-up and in Vitro Studies for Ophthalmic Nutraceutical Applications. Antioxidants.

[178] Emanuel N., Sao K., Kaushik A. (2018). Bioactive Compounds, Antioxidant Properties, and Metal Content Studies of Guava Fruit By-Products for Value Added Processing. Braz. J. Anal. Chem..

[179] Tapal A., Vegarud G.E., Sreedhara A., Kaul Tiku P. (2019). Nutraceutical Protein Isolate from Pigeon Pea (*Cajanus cajan*) Milling Waste by-Product: Functional Aspects and Digestibility. Food Funct..

[180] Tenore G.C., Caruso D., D’avino M., Buonomo G., Caruso G., Ciampaglia R., Schiano E., Maisto M., Annunziata G., Novellino E. (2020). A Pilot Screening of Agro-Food Waste Products as Sources of Nutraceutical Formulations to Improve Simulated Postprandial Glycaemia and Insulinaemia in Healthy Subjects. Nutrients.

[181] Bellumori M., De Marchi L., Mainente F., Zanoni F., Cecchi L., Innocenti M., Mulinacci N., Zoccatelli G. (2021). A By-Product from Virgin Olive Oil Production (Pâté) Encapsulated by Fluid Bed Coating: Evaluation of the Phenolic Profile after Shelf-Life Test and in Vitro Gastrointestinal Digestion. Int. J. Food Sci. Technol..

[182] Buzzi R., Gugel I., Costa S., Molesini S., Boreale S., Baldini E., Marchetti N., Vertuani S., Pinelli P., Urciuoli S. (2023). Up-Cycling of *Olea europaea* L. Ancient Cultivars Side Products: Study of a Combined Cosmetic–Food Supplement Treatment Based on Leaves and Olive Mill Wastewater Extracts. Life.

[183] Picerno P., Crascì L., Iannece P., Esposito T., Franceschelli S., Pecoraro M., Giannone V., Panico A.M., Aquino R.P., Lauro M.R. (2023). A Green Bioactive By-Product Almond Skin Functional Extract for Developing Nutraceutical Formulations with Potential Antimetabolic Activity. Molecules.

[184] Sánchez-Quezada V., Gaytán-Martínez M., Recio I., Loarca-Piña G. (2023). Avocado Seed By-Product Uses in Emulsion-Type Ingredients with Nutraceutical Value: Stability, Cytotoxicity, Nutraceutical Properties, and Assessment of In Vitro Oral-Gastric Digestion. Food Chem..

[185] Castangia I., Corrias F., Leyva Jiménez F.J., Aroffu M., Fulgheri F., Perra M., Atzei A., del Giudice A., Zengin G., Ak G. (2024). Formulation and Testing of Cutting-Edge Food Supplements Tailored for Glucose and Oxidation Controlling, Converting Artichoke By-Products in Inulin-Rich Antioxidant Phytocomplex Loaded into Zein Liposomes. Food Biosci..

[186] Grabauskaitė R., Jūrienė L., Pukalskienė M., Šipailienė A., Skurkienė R., Venskutonis P.R. (2024). Isolation of Valuable Substances from Berry Seeds and Pomace by the Green High-Pressure Methods, Their Evaluation and Application in Cosmetic Creams. Ind. Crops Prod..

[187] Maccarronello A.E., Cardullo N., Silva A.M., Di Francesco A., Costa P.C., Rodrigues F., Muccilli V. (2024). Unlocking the Nutraceutical Potential of *Corylus avellana* L. Shells: Microwave-Assisted Extraction of Phytochemicals with Antiradical and Anti-Diabetic Properties. J. Sci. Food Agric..

[188] Mehta S.K., Jafari S., Shiekh K.A., Gulzar S., Assatarakul K. (2024). Sustainable Ultrasound-Assisted Extraction and Encapsulation of Phenolic Compounds from Sacha Inchi Shell for Future Application. Sustainability.

[189] Ilgaz C., Casula L., Sarais G., Schlich M., Dessì D., Cardia M.C., Sinico C., Kadiroglu P., Lai F. (2025). Proniosomal Encapsulation of Olive Leaf Extract for Improved Delivery of Oleuropein: Towards the Valorization of an Agro-Industrial Byproduct. Food Chem..

[190] Mello V.C., de Brito G.O., Radicchi M.A., Florêncio I., Piau T.B., Ferreira E.A., de Azevedo Chang L.F., Silveira A.P., Simões M.M., de Paiva K.L.R. (2025). Advanced Solubilization of Brazilian Cerrado Byproduct Extracts Using Green Nanostructured Lipid Carriers and NaDESs for Enhanced Antioxidant Potentials. Antioxidants.

[191] Schiano E., Piccolo V., Novellino E., Maisto M., Iannuzzo F., Summa V., Tenore G.C. (2022). Thinned Nectarines, an Agro-Food Waste with Antidiabetic Potential: HPLC-HESI-MS/MS Phenolic Characterization and In Vitro Evaluation of Their Beneficial Activities. Foods.

[192] Di Lorenzo R., Castaldo L., Sessa R., Ricci L., Vardaro E., Izzo L., Grosso M., Ritieni A., Laneri S. (2024). Chemical Profile and Promising Applications of *Cucurbita pepo* L. Flowers. Antioxidants.

[193] Chauhan S., Pandit N.K., Mohanty A., Meena S.S. (2023). Resource Recovery of Bioactive Compounds from Food Waste and Their Diverse Industrial Applications. Biomass Convers. Biorefinery.

[194] Bala S., Garg D., Sridhar K., Inbaraj B.S., Singh R., Kamma S., Tripathi M., Sharma M. (2023). Transformation of Agro-Waste into Value-Added Bioproducts and Bioactive Compounds: Micro/Nano Formulations and Application in the Agri-Food-Pharma Sector. Bioengineering.

[195] Narita K., Hisamoto M., Okuda T., Takeda S. (2011). Differential Neuroprotective Activity of Two Different Grape Seed Extracts. PLoS ONE.

[196] Pasinetti G.M., Ksiezak-Reding H., Santa-Maria I., Wang J., Ho L. (2010). Development of a Grape Seed Polyphenolic Extract with Anti-Oligomeric Activity as a Novel Treatment in Progressive Supranuclear Palsy and Other Tauopathies. J. Neurochem..

[197] Loureiro J.A., Andrade S., Duarte A., Neves A.R., Queiroz J.F., Nunes C., Sevin E., Fenart L., Gosselet F., Coelho M.A.N. (2017). Resveratrol and Grape Extract-Loaded Solid Lipid Nanoparticles for the Treatment of Alzheimer’s Disease. Molecules.

[198] El-Nashar H.A.S., Abbas H., Zewail M., Noureldin M.H., Ali M.M., Shamaa M.M., Khattab M.A., Ibrahim N. (2022). Neuroprotective Effect of Artichoke-Based Nanoformulation in Sporadic Alzheimer’s Disease Mouse Model: Focus on Antioxidant, Anti-Inflammatory, and Amyloidogenic Pathways. Pharmaceuticals.

[199] Mounir R., Alshareef W.A., El Gebaly E.A., El-Haddad A.E., Ahmed A.M.S., Mohamed O.G., Enan E.T., Mosallam S., Tripathi A., Selim H.M.R.M. (2023). Unlocking the Power of Onion Peel Extracts: Antimicrobial and Anti-Inflammatory Effects Improve Wound Healing through Repressing Notch-1/NLRP3/Caspase-1 Signaling. Pharmaceuticals.

[200] Panda J., Mishra A.K., Mohanta Y.K., Patowary K., Rauta P.R., Mishra B. (2024). Exploring Biopolymer for Food and Pharmaceuticals Application in the Circular Bioeconomy: An Agro-Food Waste-to-Wealth Approach. Waste Biomass Valorization.

[201] Kučuk N., Primožič M., Kotnik P., Knez Ž., Leitgeb M. (2024). Mango Peels as an Industrial By-Product: A Sustainable Source of Compounds with Antioxidant, Enzymatic, and Antimicrobial Activity. Foods.

[202] Nguyen H.T., Miyamoto A., Hoang H.T., Vu T.T.T., Pothinuch P., Nguyen H.T.T. (2024). Effects of Maturation on Antibacterial Properties of Vietnamese Mango (*Mangifera indica*) Leaves. Molecules.

[203] Al-Naymi H.A.S., Mahmoudi E., Kamil M.M., Almajidi Y.Q., Al-Musawi M.H., Mohammadzadeh V., Ghorbani M., Mortazavi Moghadam F. (2024). A Novel Designed Nanofibrous Mat Based on Hydroxypropyl Methyl Cellulose Incorporating Mango Peel Extract for Potential Use in Wound Care System. Int. J. Biol. Macromol..

[204] Veeruraj A., Liu L., Zheng J., Wu J., Arumugam M. (2019). Evaluation of Astaxanthin Incorporated Collagen Film Developed from the Outer Skin Waste of Squid Doryteuthis Singhalensis for Wound Healing and Tissue Regenerative Applications. Mater. Sci. Eng. C.

[205] Ferreira D.F., da Silva T.M., de Melo R.C.G., Bastos K.A., Ucella-Filho J.G.M., Severi J.A., Villanova J.C.O., Resende J.A. (2024). Development of a Gel Formulation with Pomegranate Peel Extract (*Punica granatum* L.) for Antimicrobial and Wound Healing Action. S. Afr. J. Bot..

[206] Mahabeer G., Jin S. (2024). Upcycling Food Waste into Biomaterials Applicable to Medical Products. Sustainability.

[207] Shahzad A., Khan A., Afzal Z., Umer M.F., Khan J., Khan G.M. (2019). Formulation Development and Characterization of Cefazolin Nanoparticles-Loaded Cross-Linked Films of Sodium Alginate and Pectin as Wound Dressings. Int. J. Biol. Macromol..

[208] Cui X., Lee J., Ng K.R., Chen W.N. (2021). Food Waste Durian Rind-Derived Cellulose Organohydrogels: Toward Anti-Freezing and Antimicrobial Wound Dressing. ACS Sustain. Chem. Eng..

[209] Yu N., Wang X., Ning F., Jiang C., Li Y., Peng H., Xiong H. (2019). Development of Antibacterial Pectin from *Akebia trifoliata* Var. Australis Waste for Accelerated Wound Healing. Carbohydr. Polym..

[210] Zhang Y., Pham H.M., Tran S.D. (2024). The Chicken Egg: An Advanced Material for Tissue Engineering. Biomolecules.

[211] Mensah R.A., Jo S.B., Kim H., Park S.M., Patel K.D., Cho K.J., Cook M.T., Kirton S.B., Hutter V., Sidney L.E. (2021). The Eggshell Membrane: A Potential Biomaterial for Corneal Wound Healing. J. Biomater. Appl..

[212] Chuysinuan P., Nooeaid P., Thanyacharoen T., Techasakul S., Pavasant P., Kanjanamekanant K. (2021). Injectable Eggshell-Derived Hydroxyapatite-Incorporated Fibroin-Alginate Composite Hydrogel for Bone Tissue Engineering. Int. J. Biol. Macromol..

[213] Nayak S.K., Baliyarsingh B., Singh A., Mannazzu I., Mishra B.B. (2022). Advances in Agricultural and Industrial Microbiology: Applications of Microbes for Sustainable Agriculture and In-Silico Strategies.

[214] Perwez M., Al Asheh S. (2025). Valorization of Agro-Industrial Waste through Solid-State Fermentation: Mini Review. Biotechnol. Rep..

[215] Asagbra A.E., Sanni A.I., Oyewole O.B. (2005). Solid-State Fermentation Production of Tetracycline by Streptomyces Strains Using Some Agricultural Wastes as Substrate. World J. Microbiol. Biotechnol..

[216] Mahalaxmi Y., Sathish T., Subba Rao C., Prakasham R.S. (2010). Corn Husk as a Novel Substrate for the Production of Rifamycin B by Isolated *Amycolatopsis* sp. RSP 3 under SSF. Process Biochem..

[217] El-Housseiny G.S., Ibrahim A.A., Yassien M.A., Aboshanab K.M. (2021). Production and Statistical Optimization of Paromomycin by Streptomyces Rimosus NRRL 2455 in Solid State Fermentation. BMC Microbiol..

[218] Kalaiyarasi M., Ahmad P., Vijayaraghavan P. (2020). Enhanced Production Antibiotics Using Green Gram Husk Medium by *Streptomyces* sp. SD1 Using Response Surface Methodology. J. King Saud Univ.—Sci..

[219] Espro C., Paone E., Mauriello F., Gotti R., Uliassi E., Bolognesi M.L., Rodríguez-Padrón D., Luque R. (2021). Sustainable Production of Pharmaceutical, Nutraceutical and Bioactive Compounds from Biomass and Waste. Chem. Soc. Rev..

[220] Zhou S., Cao Y., Shan F., Huang P., Yang Y., Liu S. (2023). Analyses of Chemical Components and Their Functions in Single Species Plant-Derived Exosome like Vesicle. TrAC—Trends Anal. Chem..

[221] Buratta S., Latella R., Chiaradia E., Salzano A.M., Tancini B., Pellegrino R.M., Urbanelli L., Cerrotti G., Calzoni E., Alabed H.B.R. (2024). Characterization of Nanovesicles Isolated from Olive Vegetation Water. Foods.

[222] Nemati M., Singh B., Mir R.A., Nemati M., Babaei A., Ahmadi M., Rasmi Y., Golezani A.G., Rezaie J. (2022). Plant-Derived Extracellular Vesicles: A Novel Nanomedicine Approach with Advantages and Challenges. Cell Commun. Signal..

[223] Veerman R.E., Teeuwen L., Czarnewski P., Güclüler Akpinar G., Sandberg A.S., Cao X., Pernemalm M., Orre L.M., Gabrielsson S., Eldh M. (2021). Molecular Evaluation of Five Different Isolation Methods for Extracellular Vesicles Reveals Different Clinical Applicability and Subcellular Origin. J. Extracell. Vesicles.

[224] Reichert C.L., Bugnicourt E., Coltelli M.B., Cinelli P., Lazzeri A., Canesi I., Braca F., Martínez B.M., Alonso R., Agostinis L. (2020). Bio-Based Packaging: Materials, Modifications, Industrial Applications and Sustainability. Polymers.

[225] Álvarez-Chávez C.R., Edwards S., Moure-Eraso R., Geiser K. (2012). Sustainability of Bio-Based Plastics: General Comparative Analysis and Recommendations for Improvement. J. Clean. Prod..

[226] Fusco R., Siracusa R., Genovese T., Cuzzocrea S., Di Paola R. (2020). Focus on the Role of NLRP3 Inflammasome in Diseases. Int. J. Mol. Sci..

[227] Raza Z.A., Abid S., Banat I.M. (2018). Polyhydroxyalkanoates: Characteristics, Production, Recent Developments and Applications. Int. Biodeterior. Biodegrad..

[228] Spierling S., Knüpffer E., Behnsen H., Mudersbach M., Krieg H., Springer S., Albrecht S., Herrmann C., Endres H.J. (2018). Bio-Based Plastics—A Review of Environmental, Social and Economic Impact Assessments. J. Clean. Prod..

[229] Jõgi K., Bhat R. (2020). Valorization of Food Processing Wastes and By-Products for Bioplastic Production. Sustain. Chem. Pharm..

[230] Nazrin A., Sapuan S.M., Ilyas R.A., Hawanis H.S.N., Khalina A., Jumaidin R., Asyraf M.R.M., Nurazzi N.M., Norrrahim M.N.F., Rajeshkumar L. (2023). Introduction to Bio-Based Packaging Materials. Phys. Sci. Rev..

[231] Garrison T.F., Murawski A., Quirino R.L. (2016). Bio-Based Polymers with Potential for Biodegradability. Polymers.

[232] Versino F., Ortega F., Monroy Y., Rivero S., López O.V., García M.A. (2023). Sustainable and Bio-Based Food Packaging: A Review on Past and Current Design Innovations. Foods.

[233] Yuvaraj D., Iyyappan J., Gnanasekaran R., Ishwarya G., Harshini R.P., Dhithya V., Chandran M., Kanishka V., Gomathi K. (2021). Advances in Bio Food Packaging—An Overview. Heliyon.

[234] Shen Y., Seidi F., Ahmad M., Liu Y., Saeb M.R., Akbari A., Xiao H. (2023). Recent Advances in Functional Cellulose-Based Films with Antimicrobial and Antioxidant Properties for Food Packaging. J. Agric. Food Chem..

[235] García-Guzmán L., Cabrera-Barjas G., Soria-Hernández C.G., Castaño J., Guadarrama-Lezama A.Y., Rodríguez Llamazares S. (2022). Progress in Starch-Based Materials for Food Packaging Applications. Polysaccharides.

[236] Fatima S., Khan M.R., Ahmad I., Sadiq M.B. (2024). Recent Advances in Modified Starch Based Biodegradable Food Packaging: A Review. Heliyon.

[237] Santhosh R., Ahmed J., Thakur R., Sarkar P. (2024). Starch-Based Edible Packaging: Rheological, Thermal, Mechanical, Microstructural, and Barrier Properties—A Review. Sustain. Food Technol..

[238] Zhao D., Zhang X., Zhang Y., Xu E., Yan S., Xu H., Li M. (2024). Recent Advances in the Fabrication, Characterization and Application of Starch-Based Materials for Active Food Packaging: Hydrogels and Aerogels. Sustain. Food Technol..

[239] Forte J., Hanieh P.N., Poerio N., Olimpieri T., Ammendolia M.G., Fraziano M., Fabiano M.G., Marianecci C., Carafa M., Bordi F. (2023). Mucoadhesive Rifampicin-Liposomes for the Treatment of Pulmonary Infection by *Mycobacterium abscessus*: Chitosan or ε-Poly-L-Lysine Decoration. Biomolecules.

[240] Wang H., Qian J., Ding F. (2018). Emerging Chitosan-Based Films for Food Packaging Applications. J. Agric. Food Chem..

[241] Wrońska N., Katir N., Nowak-Lange M., El Kadib A., Lisowska K. (2023). Biodegradable Chitosan-Based Films as an Alternative to Plastic Packaging. Foods.

[242] Luo Q., Hossen M.A., Zeng Y., Dai J., Li S., Qin W., Liu Y. (2022). Gelatin-Based Composite Films and Their Application in Food Packaging: A Review. J. Food Eng..

[243] de Vargas V.H., Marczak L.D.F., Flôres S.H., Mercali G.D. (2023). Morphology and Functional Properties of Gelatin-Based Films Modified by UV Radiation and Bacterial Cellulose Nanofibers. J. Food Process Eng..

[244] Yu M., Hou Y., Zheng L., Han Y., Wang D. (2023). Soy Protein Isolate-Based Active Films Functionalized with Zanthoxylum Bungeanum by-Products: Effects on Barrier, Mechanical, Antioxidant and Cherry Tomato Preservation Performance. Int. J. Biol. Macromol..

[245] Abang S., Wong F., Sarbatly R., Sariau J., Baini R., Besar N.A. (2023). Bioplastic Classifications and Innovations in Antibacterial, Antifungal, and Antioxidant Applications. J. Bioresour. Bioprod..

[246] García-Juárez A., Garzón-García A.M., Ramos-Enríquez J.R., Tapia-Hernández J.A., Ruiz-Cruz S., Canizales-Rodríguez D.F., Del-Toro-Sánchez C.L., Rodríguez-Félix F., Ocaño-Higuera V.M., Ornelas-Paz J.d.J. (2024). Evaluation of Antioxidant and Antibacterial Activity of Gelatin Nanoparticles with Bitter Orange Peel Extract for Food Applications. Foods.

[247] Arafat Y., Altemimi A., Pratap-Singh A., Badwaik L.S. (2021). Active Biodegradable Films Based on Sweet Lime Peel Residue and Its Effect on Quality of Fish Fillets. Polymers.

[248] Fiorentini C., Garrido G.D., Bassani A., Cortimiglia C., Zaccone M., Montalbano L., Martinez-Nogues V., Cocconcelli P.S., Spigno G. (2022). Citrus Peel Extracts for Industrial-Scale Production of Bio-Based Active Food Packaging. Foods.

[249] Arslan D., Tuccitto N., Auditore A., Licciardello A., Marletta G., Riolo M., La Spada F., Conti Taguali S., Calpe J., Meca G. (2024). Chitosan-Based Films Grafted with Citrus Waste-Derived Antifungal Agents: An Innovative and Sustainable Approach to Enhance Post-Harvest Preservation of Citrus Fruit. Int. J. Biol. Macromol..

[250] Masssijaya S.Y., Lubis M.A.R., Nissa R.C., Nurhamiyah Y., Nugroho P., Antov P., Lee S.H., Papadopoulos A.N., Kusumah S.S., Karlinasari L. (2023). Utilization of Spent Coffee Grounds as a Sustainable Resource for the Synthesis of Bioplastic Composites with Polylactic Acid, Starch, and Sucrose. J. Compos. Sci..

[251] Mendes J.F., Martins J.T., Manrich A., Sena Neto A.R., Pinheiro A.C.M., Mattoso L.H.C., Martins M.A. (2019). Development and Physical-Chemical Properties of Pectin Film Reinforced with Spent Coffee Grounds by Continuous Casting. Carbohydr. Polym..

[252] Dordevic D., Dordevic S., Abdullah F.A.A., Mader T., Medimorec N., Tremlova B., Kushkevych I. (2023). Edible/Biodegradable Packaging with the Addition of Spent Coffee Grounds Oil. Foods.

[253] Amiri Samani S., PourvatanDoust S., Savarolyia M., Aboutalebzadeh S., Khezri M., Kazemi M., Khodaiyan F., Hosseini S.S. (2025). Valorization of Red Grape Pomace for Sustainable Food Packaging: Development of Pectin/Kidney Bean Protein Based Biocomposite Films Enriched with Grape Pomace Polyphenols. Food Hydrocoll..

[254] Gubitosa J., Rizzi V., Marasciulo C., Maggi F., Caprioli G., Mustafa A.M., Fini P., De Vietro N., Aresta A.M., Cosma P. (2023). Realizing Eco-Friendly Water-Resistant Sodium-Alginate-Based Films Blended with a Polyphenolic Aqueous Extract from Grape Pomace Waste for Potential Food Packaging Applications. Int. J. Mol. Sci..

[255] Gowman A., Wang T., Rodriguez-Uribe A., Mohanty A.K., Misra M. (2018). Bio-Poly(Butylene Succinate) and Its Composites with Grape Pomace: Mechanical Performance and Thermal Properties. ACS Omega.

[256] Diaz-Herrera R., Alvarez-Pérez O.B., Ventura-Sobrevilla J., Ascacio-Valdés A., Aguilar-Gonzalez M.A., Buenrostro-Figueroa J., Aguilar C.N. (2023). Pomegranate Peel Polyphenols as an Antioxidant Additive for the Development and Characterization of a New Active Pectin Edible Film. eFood.

[257] Nabeel Ahmad H., Yong Y., Wang S., Munawar N., Zhu J. (2024). Development of Novel Carboxymethyl Cellulose/Gelatin-Based Edible Films with Pomegranate Peel Extract as Antibacterial/Antioxidant Agents for Beef Preservation. Food Chem..

[258] Lammi S., Le Moigne N., Djenane D., Gontard N., Angellier-Coussy H. (2018). Dry Fractionation of Olive Pomace for the Development of Food Packaging Biocomposites. Ind. Crops Prod..

[259] Fiorentini C., Leni G., de Apodaca E.D., Fernández-de-Castro L., Rocchetti G., Cortimiglia C., Spigno G., Bassani A. (2024). Development of Coated PLA Films Containing a Commercial Olive Leaf Extract for the Food Packaging Sector. Antioxidants.

[260] Chabni A., Bañares C., Sanchez-Rey I., Torres C.F. (2025). Active Biodegradable Packaging Films Based on the Revalorization of Food-Grade Olive Oil Mill By-Products. Appl. Sci..

[261] Apicella A., Adiletta G., Di Matteo M., Incarnato L. (2019). Valorization of Olive Industry Waste Products for Development of New Eco-Sustainable, Multilayer Antioxidant Packaging for Food Preservation. Chem. Eng. Trans..

